# Structural Variations Affecting Genes and Transposable Elements of Chromosome 3B in Wheats

**DOI:** 10.3389/fgene.2020.00891

**Published:** 2020-08-18

**Authors:** Romain De Oliveira, Hélène Rimbert, François Balfourier, Jonathan Kitt, Emeric Dynomant, Jan Vrána, Jaroslav Doležel, Federica Cattonaro, Etienne Paux, Frédéric Choulet

**Affiliations:** ^1^Université Clermont Auvergne, INRAE, GDEC, Clermont-Ferrand, France; ^2^Institute of Experimental Botany of the Czech Academy of Sciences, Centre of the Region Haná for Biotechnological and Agricultural Research, Olomouc, Czechia; ^3^IGA, Udine, Italy

**Keywords:** bread wheat, genome evolution, structural variations, copy number variations, transposable elements, *Triticum*

## Abstract

Structural variations (SVs) such as copy number and presence–absence variations are polymorphisms that are known to impact genome composition at the species level and are associated with phenotypic variations. In the absence of a reference genome sequence, their study has long been hampered in wheat. The recent production of new wheat genomic resources has led to a paradigm shift, making possible to investigate the extent of SVs among cultivated and wild accessions. We assessed SVs affecting genes and transposable elements (TEs) in a *Triticeae* diversity panel of 45 accessions from seven tetraploid and hexaploid species using high-coverage shotgun sequencing of sorted chromosome 3B DNA and dedicated bioinformatics approaches. We showed that 23% of the genes are variable within this panel, and we also identified 330 genes absent from the reference accession Chinese Spring. In addition, 60% of the TE-derived reference markers were absent in at least one accession, revealing a high level of intraspecific and interspecific variability affecting the TE space. Chromosome extremities are the regions where we observed most of the variability, confirming previous hypotheses made when comparing wheat with the other grasses. This study provides deeper insights into the genomic variability affecting the complex *Triticeae* genomes at the intraspecific and interspecific levels and suggests a phylogeny with independent hybridization events leading to different hexaploid species.

## Introduction

Bread wheat is one of the most consumed crops in the world (Hawkesford et al., 2013), and a better knowledge of the extent of genomic diversity among wheat and its wild relatives is needed to meet the challenge of ensuring food security in the context of climate change and sustainability of agriculture. However, genomic studies in wheat are facing intrinsic genome complexity due to allohexaploidy (2*n* = 6× = AABBDD), 85% of transposable elements (TEs), and a large genome size (∼15.5 Gb). Bread wheat originated from two interspecific hybridization events: the first one occurred ∼800,000 years ago between *Triticum urartu* (AA) and a species close to *Aegilops speltoides* (BB); the second one occurred ∼10,000 years ago between *Triticum turgidum* (AABB) and *Aegilops tauschii* (DD) (Marcussen et al., 2014). A reference sequence of the complete bread wheat genome of cv. Chinese Spring (CS) has been produced recently by the International Wheat Genome Sequencing Consortium, revealing 107,891 gene models and around 4 million TEs (IWGSC, 2018). Chromosome 3B is the largest of the 21 wheat chromosomes and, thus, the only chromosome that can be isolated individually from any wheat cultivar using flow cytometric sorting (Doležel et al., 2007). This is why chromosome 3B was established as the model to study the wheat genome. Its analysis highlighted a structural and functional partitioning, and this particular organization was then found to be common to all wheat and barley chromosomes (Choulet et al., 2014; Mascher et al., 2017; IWGSC, 2018). Indeed, chromosomal extremities are the regions where most of recombination events occur, are enriched in genes, and are depleted in TEs (Choulet et al., 2014; Daron et al., 2014). They are also enriched in non-syntenic and recently duplicated genes whose functions are related to adaptation (Glover et al., 2015; Pingault et al., 2015). These conclusions were drawn through comparisons with rice, maize, *Brachypodium*, and sorghum, but little is known about the extent of structural variability among *Triticeae* and at the intraspecific level.

Structural variations (SVs) are genomic polymorphisms that could originate from insertion, duplication, deletion, translocation, or inversion (Alkan et al., 2011). Insertions/duplications and deletions lead to polymorphisms affecting the gene content called copy-number variations (CNVs) and presence–absence variations (PAVs). In plants, such SVs have been associated with a diversity of phenotypes for major traits like photoperiod sensitivity and vernalization (Sutton et al., 2007; Cook et al., 2012; Maron et al., 2013). In wheat, CNVs affect *Ppd-B1*, *Vrn-A1*, and *Rht-D1*, the key genes controlling photoperiod, vernalization, and dwarfism, respectively (Díaz et al., 2012; Li et al., 2012; Würschum et al., 2015). Previous studies attempted to assess the landscape of SVs at large scales, but the complexity of the genome limits our ability to identify them accurately. Eighty-five CNVs and five PAVs were detected between two tetraploid wheats based on the analysis of 3497 cDNAs (Saintenac et al., 2011). In 2017, Montenegro et al. analyzed resequencing data from a panel of 18 bread wheat accessions and estimated a pangenome of ∼140,500 genes (Montenegro et al., 2017). A comparative sequence analysis of chromosome 2D between two distant accessions of *T. aestivum* revealed that 13% and 20% of gene models are specific to each other (Thind et al., 2018). Comparison between Chinese Spring, Synthetic w7984, and Opata highlighted that large (at least 100 kb) SVs were more abundant in distal regions (Gutierrez-Gonzalez et al., 2019).

Little is known about SVs affecting wheat TEs. However, they account for 85% of the genome and, in this regard, are the major drivers of the wheat genome dynamics. A recent study showed that the vast majority of TEs that are present in the hexaploid genome inserted after A, B, and D genomes diverged from a common ancestor ∼3 Mya, but before the polyploidization events (Wicker et al., 2018). This resulted in a nearly complete TE turnover making A–B–D homeologous loci completely different in terms of TEs. However, the global genome architecture remained stable, suggesting a role of TEs in maintaining a constrained genome architecture. Although polyploidization did not trigger massive TE activation, the TE turnover is still an ongoing process at the origin of genome diversification of di-, tetra-, and hexaploid wheats (Keidar-Friedman et al., 2018; Arystanbekkyzy et al., 2019).

Here, we used chromosome sorting combined with shotgun sequencing in order to decipher the extent of SVs affecting chromosome 3B using a diverse panel of 45 *Triticum* accessions from seven species. We established robust methods based on short-read mapping to accurately call PAVs and CNVs affecting genes and TEs while preventing from the complexity due to polyploidy. We show that 23% of the genes are variable among this diverse panel and more than half of the TEs is affected by PAVs. This gave us the most detailed and accurate view of the landscape of SVs in such a complex genome and confirmed that distal chromosomal regions are privileged regions for rapid diversification.

## Results

### Resequencing Chromosome 3B From 45 Triticeae Accessions and Quality Assessment

To study the extent of SVs in the wheat genome, chromosome 3B (chr3B) was flow-sorted from 45 accessions belonging to seven wheat species: hexaploid *T. aestivum* (*Tae*), *T. spelta* (*Tsp*), *T. macha* (*Tma*), and tetraploid *T. durum* (*Tdu*), *T. dicoccoides* (*Tde*), *T. dicoccum* (*Tdi*), and *T. carthlicum* (*Tca*) (Table 1). Our panel also included one hexaploid synthetic accession (Synthetic w7984), formed by hybridizing a tetraploid wheat *T. turgidum* L. subsp. *durum* var “Altar 84” (AABB) with the diploid goat grass *Aegilops tauschii* (219; CIGM86.940, DD genotype) (Sorrells et al., 2011).

**TABLE 1:** List of accessions and metrics of resequencing samples and mapping data.

						Mapped reads (%)			
Species (ploidy)	ERGE id	Accession name	Country	Status	No. raw reads	Whole genome	chr3B	Depth of coverage (x)	Coverage (%)
*Tae* (6×)	2135	Chinese Spring	China	L	844,814,494	97.9	87.5	85.2	96.2
	6575	Sirazaiabarigi 2	Japan	NA	319,563,282	99.1	88.5	29.5	91.9
	5116	Nanking_NO25	China	L	319,034,032	99.5	87.3	23.5	91.5
	3176	Fukuhokomugi	Japan	M	264,900,600	90.2	81.9	19.6	91.9
	5401	NZ(81)P43	New Zealand	M	258,494,970	99.3	86.4	21.1	87.7
	4477	M45/66	Argentina	NA	303,699,738	98.1	85.3	34.5	85.4
	5096	N67M2	Israel	NA	296,790,036	98.7	84.7	32.2	91.2
	5108	Nachipundo	Nepal	NA	304,170,350	98.4	83.8	32.3	89.0
	5536	Oulianowska	Russia	M	189,787,528	99.2	88.3	15.2	88.7
	964	Arche	France	M	170,560,868	98.8	87.0	14.0	86.9
	13445	Volt	Hungary	M	327,525,574	98.7	86.0	27.8	89.1
	2475	Detenicka Cervena	Czech	L	231,846,258	99.0	87.2	17.6	92.1
	1899	Cerealor	France	M	276,070,518	98.9	87.9	20.3	88.4
	6027	Recital	France	M	236,586,016	97.1	87.1	25.7	89.0
	5425	Odessa Expsta21821	Portugal	NA	230,518,384	99.1	86.5	18.6	85.0
	6047	Redman	Canada	T	278,370,090	99.1	85.1	21.6	89.6
	4634	Marquis	Canada	T	429,295,104	99.5	85.6	35.2	89.0
	13811	Opata 85	Mexico	M	226,031,606	98.6	82.8	25.1	88.1
	3650	Hope	United States	T	417,393,134	99.4	75.2	32.0	87.6
	6986	Tom Thumb	China	M	395,383,156	98.6	83.2	32.0	87.7
*Tma* (6×)	24175	24175	Georgia	L	414,689,254	99.6	79.0	37.6	88.2
	24174	24174	Georgia	L	336,054,982	99.5	83.0	31.6	87.9
	24176	24176	Georgia	L	335,531,572	99.5	77.1	30.3	87.7
*Tsp* (6×)	9028	EPEAUTRE 3 ROUX	France	L	417,456,256	99.4	80.8	36.7	87.6
	2774	EPEAUTRE DE L’AVEYRON	France	L	353,258,002	99.5	78.2	32.9	87.3
	2776	EPEAUTRE NOIR BARBU VELU	France	L	400,907,560	99.3	69.5	33.1	88.2
*Tdi* (4×)	33758	45383	Bulgaria	L	426,702,308	97.5	55.2	25.6	85.8
	33757	CWI17084	Iraq	L	423,862,292	99.3	80.8	38.4	85.2
	33763	45309	Slovakia	L	448,841,200	89.9	77.9	34.7	84.6
	33767	415152	Israel	L	433,236,238	99.1	73.1	30.5	85.3
*Tde* (4×)	26677	DD.100V	Hungary	L	708,950,828	99.4	78.8	59.8	88.9
	26676	DD.PSEUDO-JORDANIC.61V	Slovakia	L	439,492,298	98.3	74.7	34.7	87.9
	26678	DD.SPONTANEO VILLOSUM 117V	Chile	L	390,345,462	99.4	74.6	32.8	88.4
*Tca* (4×)	33753	251914	Georgia	L	421,283,854	99.5	80.3	33.7	87.3
	33751	94753	Georgia	L	444,667,448	99.4	82.4	39.8	87.7
	33749	94750	Georgia	L	446,411,378	99.4	80.3	34.5	87.4
	33750	94755	Georgia	L	354,525,712	99.4	63.9	25.8	87.1
*Tdu* (4×)	33800	82715	Turkey	NA	450,830,834	99.4	87.7	45.0	87.7
	33795	KUBANKA	Russia	T	424,719,626	99.3	80.9	39.0	86.6
	33796	84866	Syria	T	443,666,174	99.2	84.9	43.0	85.2
	NA	Strongfield	Canada	M	399,722,126	99.4	84.7	35.1	86.4
	NA	Kofa	United States	M	451,021,954	99.4	81.3	38.4	86.2
	28794	Langdon	United States	T	307,801,782	99.2	87.4	29.6	87.4
	NA	Svevo	Italy	M	336,828,550	99.5	85.7	34.9	87.6
Synthetic (6×)	13812	W7984 (Synthetic)	Mexico	NA	257,057,600	98.3	77.5	15.6	85.3

**Tae*, *T. aestivum*; *Tma*, *T. macha*; *Tsp*, *T. spelta*; *Tdi*, *T. dicoccum*; *Tde*, *T. dicoccoides*; *Tca*, *T. carthlicum*; *Tdu*, *T. durum*; Status L, Landrace; M, Modern; T, Traditional; NA, Missing information.*

DNA of sorted chromosomes was amplified and sequenced with Illumina HiSeq-2000, yielding between 171 and 845 million reads per sample, corresponding to an estimated sequencing depth of 43× on average (Table 1). The 45 resequencing datasets were mapped on the complete Chinese Spring (CS) genome sequence (IWGSC RefSeq_v1.0). On average, 98.6% of reads were mapped, demonstrating the absence of a major contamination in our samples (Table 1) and that sequence divergence between different *Triticum* genomes did not prevent from mapping reads accurately on the CS reference. On average, 81% of mapped reads were mapped onto the chr3B pseudomolecule. The remaining 19% are mapped onto unanchored scaffolds (chrUn; 4% of mapped reads) but also to other chromosomes, suggesting the presence of DNA from other chromosomes in the sorted DNA samples (especially chr2B whose size is close to that of chr3B; Additional file 1: Supplementary Table S1). The distribution of mapped reads along the 20 other chromosomes did not reveal any case of homeologous exchange with 3A or 3D, neither translocated from other chromosomes.

Based on the non-repeated fraction of chr3B (representing 134 Mb, see Materials and Methods), we found that 89% of the chromosome was covered, on average, per accession (Table 1). We used the Chinese Spring sample as a positive control and found that 4% of these regions were not covered, comprising 47 gene models. We discarded them from further analyses. The coverage ranged from 85 to 92% for the 44 other accessions, revealing that chr3B has not been affected by a large introgression, translocation, or deletion in any accession. No correlation was found between the coverage and depth of coverage (DoC; *R* = 0.26, *P* > 0.05), meaning that depth is high enough to avoid bias in SV detection (Additional file 1: Supplementary Figures S1–S2).

### SNP Diversity and Haplotypes Across chr3B

**FIGURE 1:**
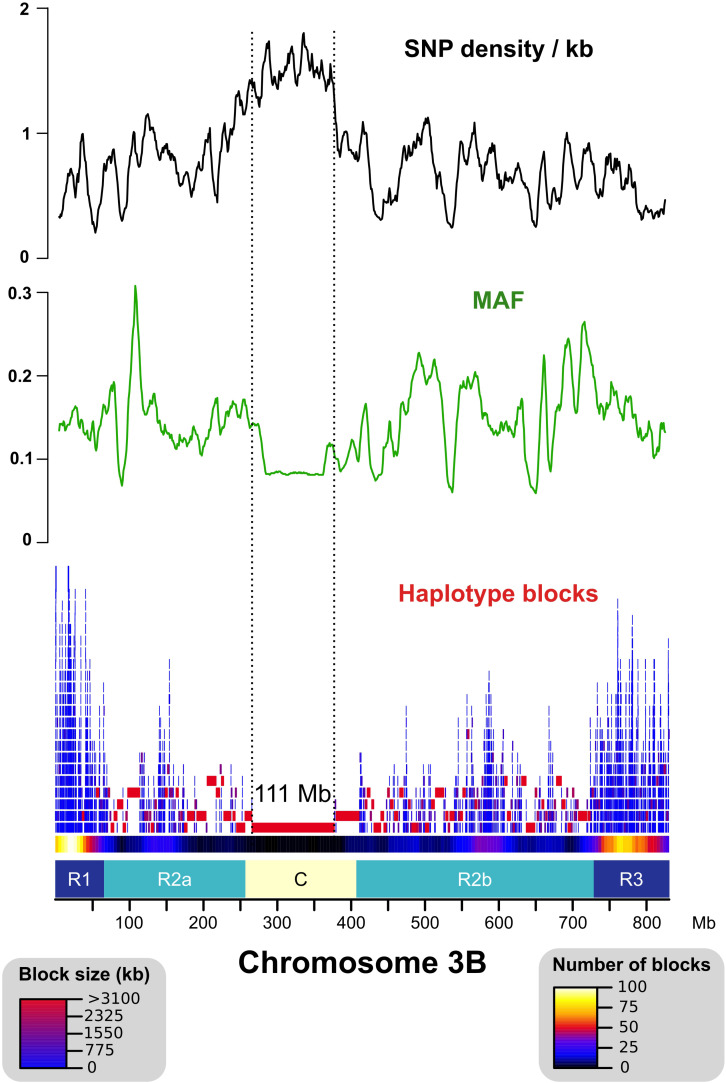
Distribution of the SNP density, minor allele frequency (MAF), and haplotype blocks across chromosome 3B. SNP density is represented as the number of SNPs per kb of uniquely mappable sequences. Distal (R1–R3), interstitial (R2a–R2b), and centromeric/pericentromeric (C) regions of chr3B are represented as defined in Choulet et al., 2014. The “C” region exhibits higher SNP density, lower MAF, and larger haplotype blocks (max = 111 Mb) than the other regions.

After read mapping, we called single-nucleotide polymorphisms (SNPs) in the non-repeated space of chr3B. In addition, we also searched for SNPs in the TE space by analyzing reads that were mapped uniquely onto markers called ISBPs (insertion site-based polymorphisms; adapted from Rimbert et al., 2018). For that, we extracted 150 bps spanning the junction between a TE and its flanking sequences at both 5′ and 3′ ends. ISBPs are unique k-mers that can be used to map reads unambiguously (Additional file 1: Supplementary Figure S3). In total, 605,443 SNPs were detected (transition/transversion ratio: 2.33). It ranged from 41,255 for East Asian *T. aestivum* to 165,932 for *T. dicoccoides*, and 74% of SNPs were shared by at least two accessions. The SNP density was higher in the centromere proximal region (called “C”) for all accessions except for the East Asian *T. aestivum* lines that were almost devoid of SNPs within a 250–Mb region encompassing the centromere (Figure 1 and Additional file 1: Supplementary Figures S4, S5). These results agree with recent results showing a clear separation between Asian and European *T. aestivum* accessions (Balfourier et al., 2019). Minor allele frequency (MAF) was lower in the C region, indicating an enrichment in rare alleles (Figure 1).

We computed haplotype blocks based on linkage disequilibrium (LD) between SNPs. This highlighted strong differences along the chromosome with large blocks in the central part of the chromosome and small blocks in the distal regions (Figure 1). This is in accordance with the recombination pattern observed for this chromosome (Choulet et al., 2014).

### Structural Variations Affecting TEs

TEs account for 84% of chromosome 3B sequence with 216,378 elements belonging to 482 families. To assess the level of variability of the TE space, we designed 309,462 ISBPs (that are uniquely mappable) representing the extremities of 211,494 TEs (i.e., 98% of all chr3B TEs, Additional file 1: Supplementary Figure S3). Read mapping revealed that 186,611 ISBPs (60%) designed from 154,012 TEs (73%) were absent in at least one accession of the panel. These results highlight the extreme variability of the TE space since more than half of the 700 Mb of TEs are not conserved among the *Triticum* genus. This variability differs strongly between individuals and species, with TE inDels ranging from 7% in *Tae_sirazaiaibarigi_2* to 24% in *Synthetic–w7984*. When only considering the three closest accessions from CS (East–Asian *T. aestivum*), inDels affect 7–8% of the TEs. Such polymorphisms were mainly shared by at least 2 accessions (155,830/186,611, 84%) and shared within each species (from 63% to 79% of shared polymorphisms, Additional file 1: Supplementary Table S2).

For 193,054 TEs (91%), we were able to design ISBPs at both 5′ and 3′ ends, therefore allowing us to trace the complete (two ends) vs partial (one end) absence of the element. Out of these, absence of both ends was observed for 38% (75,089). This revealed that SVs are mainly due to small deletions covering TEs only partially, rather than large deletions. This also revealed inDels that are due to recent TE insertions corresponding to cases where a TE is conserved among a monophyletic group while totally absent in all other accessions. We found 1,445 TEs conserved only among *T. aestivum* accessions, 574 (338 Gypsy, 60 Copia, 37 unclassified LTR-RTs, 7 LINEs, 117 DNA transposons, and 15 unclassified) conserved only among East Asian accessions, and 76 (42 retrotransposons, 30 DNA-transposons, and 4 unclassified) specific to CS. Extrapolating these values to the whole genome, one could expect roughly 29,000 new TE insertions since the hexaploidization, 10,000 years ago and 1,500 new TEs inserted in CS since it diverged, very recently, from *Tae_Nanking_No25*, *Tae_Fukuhokomugi*, and *Tae_Sirazaiabarigi_2*. We did not find any TE family contributing massively to these recent insertions, showing that no TE burst occurred recently. Thus, we conclude that the TE space is highly variable between *Triticeae* accessions, with a core-TE space restricted to 40% of the studied loci, mainly because of small deletions rather than to transposition.

In addition to the ISBP-based approach, we established a second approach in order to estimate the variations in copy number for the 482 TE families by analyzing the depth of coverage. We mapped reads on the whole CS genome, focused only on reads mapped inside TEs, and weighed read counts according to multiple mapping (see Materials and Methods). The main result is that, at the TE family and subfamily levels, no strong difference in copy number was detected for any of the 45 accessions (Additional file 1: Supplementary Figure S6). No massive amplification of any TE family was detected. The biggest difference observed here was a three-fold increase of the *Mutator* family DTM_famc6 in *Tdi_45309* compared to CS. Among *T. aestivum*, the three accessions *Nachipundo*, *Tom Thumb*, and *Hope* exhibit some differences of TE composition, although it concerns a limited number of families (Additional file 1: Supplementary Figure S6). *Tom Thumb* and *Hope* are known to originate from a hybrid cross, which might explain their slightly different TE composition (see paragraph “Phylogenetic analysis”). *Nachipundo* originates from Nepal, and it was previously shown that Nepalese accessions are clearly distinct (Horvath et al., 2009). Six gypsy subfamilies have an increased copy number in *Nachipundo* compared to CS: Danae (RLG_famc9.3), Nusif (RLG_famc4.1, RLG_famc4.2, RLG_famc4.3), RLG_famc24, and RLG_famc25.

### Structural Variations Affecting Genes and Identification of Genes Absent From CS

**FIGURE 2:**
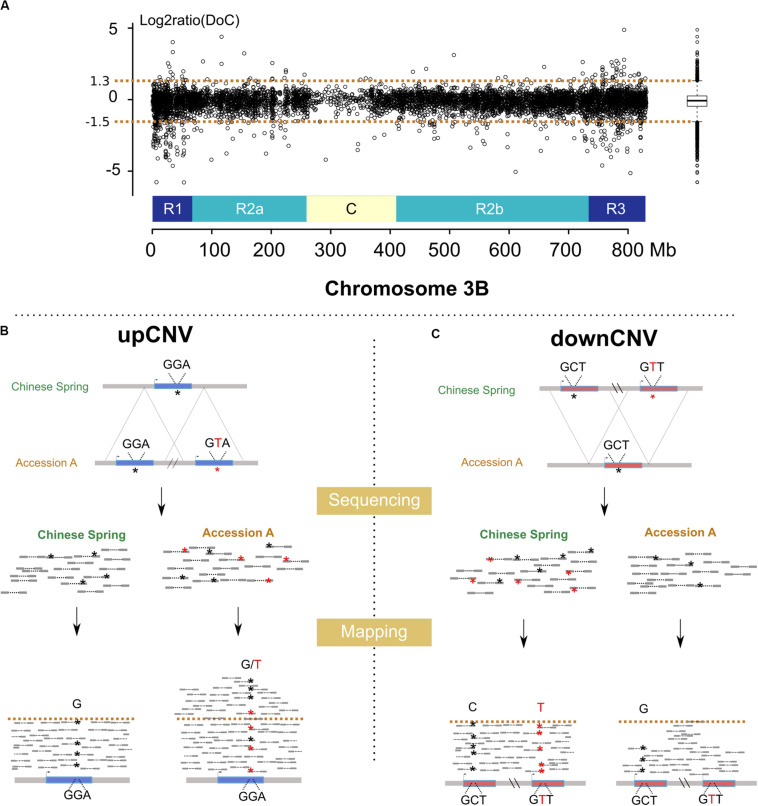
Methods developed for the detection of CNVs using resequencing data. **(A)** log2ratio of the DoC calculated across chromosome 3B for the accession *Tae_Oulianowska* compared to the reference Chinese Spring. A boxplot of the log2ratios is represented on the right, with upper and lower whiskers considered as thresholds to define up and downCNVs. Similar plots for all accessions are provided in Figures S8–15. **(B)** Detection of upCNVs based on increased DoC and presence of pseudo–heterozygous SNPs in mapped reads. Reads carrying the variant nucleotide are labeled by a red asterisk. **(C)** Detection of downCNVs based on a decreased DoC of genes duplicated in Chinese Spring (represented in red).

We analyzed the variation of the depth of coverage (DoC; Figure 2A) across genes and their surrounding low copy sequences in order to identify potential CNVs (see Materials and Methods). This led to identifying 761 genes (i.e., 13% of the 6,082 gene set) potentially affected by upCNVs (i.e., increased number of copies compared to CS). To add a layer of evidence for the existence of these upCNVs, we searched for the presence of pseudo-heterozygous SNPs in the mapping data. Indeed, since wheat accessions are highly homozygous, the presence of heterozygous SNPs supports the existence of slightly divergent paralogous copies (Figure 2B). Indeed, pseudo-heterozygous sites were highly enriched in the 761 candidates compared to other genes (34% vs 1%, respectively). By combining DoC increase and presence of pseudo-heterozygous SNPs, we ended up with 210 genes affected by upCNVs. They represent on average 21 (0.3%) genes per accession, ranging from 7 to 49, and the vast majority of them (70%) were shared between at least two accessions (Table 2 and Additional file 1: Supplementary Table S3). Among the 210 upCNVs, analysis of amino acid changes observed in duplicated gene copies revealed that 31 exhibit mutations with a strong effect (start codon loss or new stop codon), 140 have variations that only moderately affect the protein (missense variants), and 39 have only mutations that are synonymous or within UTRs. We also found 15 cases of genes with potential altered splicing.

**TABLE 2:** Number of genes affected by SVs.

	*Tde*	*Tdi*	*Tca*	*Tdu*	*Tma*	*Tsp*	*Tae (others)*	*Tae* (East Asia)	All accessions
**upCNVs**									
Unique	27	20	6	43	18	24	52	23	
Shared	21	10	11	47	13	20	60	13	
All	8	0	8	2	9	3	1	1	
Total	56	30	25	92	40	47	113	37	210
**downCNVs**									
Unique	39	51	11	23	40	31	39	36	
Shared	37	46	24	116	13	30	127	19	
All	54	30	57	25	48	56	12	37	
Total	130	127	92	164	101	117	178	92	250
**PAVs**									
Unique	61	207	29	138	36	101	127	105	
Shared	73	161	70	264	44	74	283	35	
All	54	39	126	23	103	91	5	17	
Total	188	407	225	425	183	266	415	157	781
**New genes (absent from the reference)**									
Unique	68	63	34	82	37	44	69	34	
Shared	36	44	28	114	22	34	155	17	
All	13	2	17	2	20	13	0	6	
Total	117	109	79	198	79	91	224	57	330

*The numbers of genes affected by CNVs and PAVs are indicated for each species. “Unique” indicates the number of SVs that are present in only one accession of the species. “Shared” and “All” indicate the number of SVs that are shared by at least two accessions or shared among all accessions, respectively. *Tde*, *T. dicoccoides*; *Tdi*, *T. dicoccum*; *Tca*, *T. carthlicum*; *Tdu*, *T. durum*; *Tma*, *T. macha*; *Tsp*, *T. spelta*; *Tae*, *T. aestivum*.*

For downCNVs (decrease in copy number compared to CS; Figure 2C), we first identified duplicated genes in CS. Clustering of the 6,129 CS genes revealed the presence of 443 duplicated genes belonging to 191 clusters. Among them, 250 genes (56% of the duplicated genes; 4% of the total gene set) exhibit a lower copy number in at least one accession of our panel (Table 2; Additional file 1: Supplementary Table S3), ranging from 58 for *Tae_Sirazaiabarigi_2* to 116 for *Tdu_Svevo*. Most downCNVs (88%) were common to at least two accessions (Table 2; Additional file 1: Supplementary Table S3). This analysis led to identifying two genes (TraesCS3B01G011000: putative zinc ion binding protein, and TraesCS3B01G059600: putative D-Ala-D/L-Ala epimerase) that were duplicated only in CS.

For gene PAVs, we considered a threshold of 10% of the gene length covered. In total, 781 genes were affected by PAVs, representing 14% of chr3B single-copy genes. They represent on average 145 genes (2%) per accession, ranging from 42 to 217 (0.7–3.5%; Table 2; Additional file 1: Supplementary Table S3). As expected, East Asian *T. aestivum* accessions exhibit the lower rate of variations with 75 genes (1%) showing PAVs, while *T. carthlicum* exhibit the highest: 184 (3%) genes. Only one gene is absent in all tested accessions, meaning it is specific to CS: a cupredoxin-encoding gene (TraesCS3B01G482300).

A *de novo* assembly of the unmapped reads was conducted for each sample to identify genes that are absent from the reference. We retrieved between 695 and 11,173 contigs (> 500 bps) per dataset representing between 620 kb and 7.96 Mb (<1% of chr3B size). We searched for similarity with known proteins from related plants (see Materials and Methods) and applied a clustering step to detect genes present in several accessions. Hence, we identified 330 genes (possibly pseudogenes) absent from the reference CS but present in our diversity panel and shared by at least two accessions. They represent approximately 1% of the gene repertoire (Additional file 1: Supplementary Table S3).

### The Wheat Pan- and Core-Chromosome 3B

We applied the concept of pan- and core genomes to describe the extent of variability among *Triticum* chr3B. Detection of PAVs, CNVs, and newly assembled genes revealed a pan-chromosome with 6,459 genes (6129 + 330) and a core genome of 5,134 genes, i.e., 79%. In total, 1,509 genes were variable in terms of presence/absence and copy number among the panel, i.e., 23% of the pan-chromosomes. Pairwise comparisons with CS revealed on average 293 variable genes (5%), ranging from 157 (2% for *Tae_Nanking_No25*) to 358 (6% for *Tsp_Epeautre_de_L’Aveyron*) (Additional file 1: Supplementary Table S3).

We observed that SVs more likely affect genes that were previously defined as non-syntenic (Glover et al., 2015), i.e., not conserved at a syntenic position with the related grasses (*Brachypodium*, rice, and sorghum). Gene Ontology (GO) term enrichment revealed that variable genes tend to be enriched in functions like protein phosphorylation and protein catabolic process (Table 3). It also pointed out the over-representation of functions related to pathogen resistance: 103 receptor-like kinase genes (RLKs) and 62 nucleotide-binding domain and leucine-rich-repeat-containing proteins (NLRs) are subject to CNVs in our panel. As an example, the two NLRs TraesCS3B01G081400 and TraesCS3B01G083800 are upCNVs in eight and nine accessions, respectively. While the two NLRs are single-copy genes in CS (given the criteria used for clustering, see Materials and Methods), the DoC fold change is between 2.3 and 3.5 for TraesCS3B01G081400 and between 3.0 and 6.5 for TraesCS3B01G083800. Combined with the presence of pseudo-heterozygous SNPs, it showed at least one additional copy. Another example is the case of four Flowering Locus T-like genes (two pairs of duplicates: TraesCS3B01G007400-010100 and TraesCS3B01G015600-015200) that were downCNVs, thus, single-copy genes, in 14 and 33 accessions, respectively. The well-known family of Flowering Locus T-like genes is a large family involved in the regulation of floral transition and developmental processes like fruit set, vegetative growth, stomatal control, and tuberization (Pin and Nilsson, 2012). In addition, we found another FLT-like gene (TraesCS3B01G489500) affected by upCNVs (in *Tae_nachipundo* and *Tae_n67m2*) and PAVs (in *Tea_sirazaiaibarigi*).

**TABLE 3:** GO term enrichment of the 1179 genes affected by SVs.

GO.ID	Function	No. genes	Significant	Expected	*p*-Value (fisher test)
GO:0006869	Lipid transport	23	17	3.97	2.7e-09
GO:0009664	Plant-type cell wall organization	23	14	3.97	1.0e-05
GO:0006468	Protein phosphorylation	308	77	53.1	0.0001
GO:0007275	Multicellular organism development	40	15	6.9	0.0016
GO:0006511	Ubiquitin-dependent protein catabolic process	56	17	9.66	0.0102
GO:0006412	Translation	90	24	15.52	0.0166
GO:0006559	L-Phenylalanine catabolic process	2	2	0.34	0.0297
GO:0009767	Photosynthetic electron transport chain	5	3	0.86	0.0387

We also compared the expression pattern and histone marks of variable vs core genes using the available RNAseq data produced in 15 conditions (Pingault et al., 2015) and CHIP-seq data (3-leaf stage; IWGSC, 2018; Ramírez-González et al., 2018), both for CS. Variable genes tend to be expressed at a lower level in fewer conditions and tend to be targeted by facultative repressive epigenetic mark H3K27me3 (Additional file 1: Supplementary Figure S7). Altogether, these data are consistent with previous findings and confirm that, as observed in other species, *Triticeae* variable genes tend to exhibit features of genes related to the adaptation of the plant to its environment.

### Distribution of Polymorphisms

**FIGURE 3:**
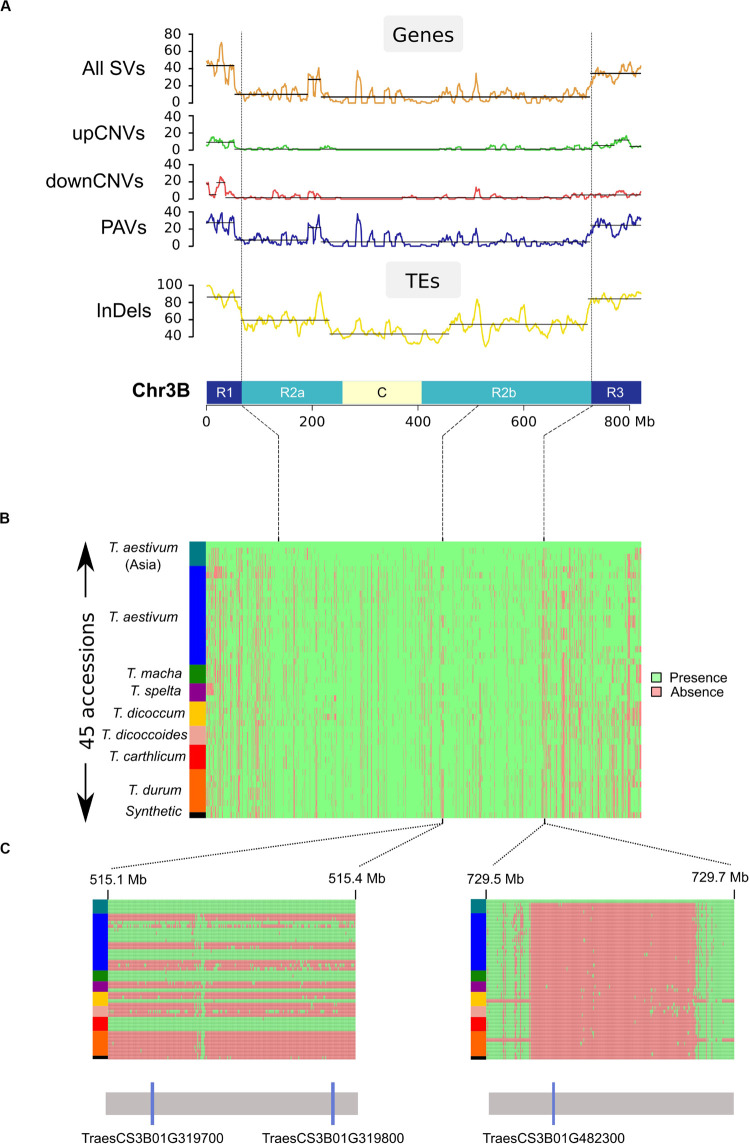
Distribution of structural variations across chromosome 3B. **(A)** Distribution of SVs affecting genes and TEs calculated in a sliding window of 10 Mb (step = 1 Mb). Chromosome 3B is represented at the bottom of the graphs with distal (R1 and R3), interstitial (R2a and R2b), and centromeric/pericentromeric **(C)** regions. **(B)** PAV matrix of the 1,593,66 studied loci across chr3B for the 45 accessions (1 line per accession). Accessions were ordered according to their phylogenetic relationships (see “Phylogenetic analysis”). Presence and absence are shown in green and red, respectively. **(C)** Zoom in view for two regions: the left region carries a pair of duplicated genes (TraesCS3B01G319700 and TraesCS3B01G319800) that are either fully absent or present in our panel. The right region carries gene TraesCS3B01G482300 present only in Chinese Spring.

We called PAVs across the non-repeated fraction of the chromosome by splitting the sequence into segments of 100 bps while excluding TEs except previously designed ISBPs. In total, this represented 1,593,666 ordered segments cumulating 134 Mb (see Materials and Methods). We then used a sliding window of 50 segments to define regions of varying size affected by a PAV (Figure 3). Between 626 and 2,372 PAVs were found for the 45 accessions. The average size of a PAV was between 29 and 70 kb. The largest region was 6 Mb absent in *Tsp_Epeautre_noir_barbu_velu* (from position 215,620,552 to 221,751,806 bps). These results highlighted the fact that most of the structural variability between these species/accessions originated from small deletion events occurring across the whole chromosome (Figure 3). We did not observe any large deletion, translocation, or introgression event in any of the tested accessions.

These data allowed us to study the distribution of SVs across the chromosome in regard to the partitioning previously mentioned for the *Triticeae* chromosomes (Choulet et al., 2014; Mascher et al., 2017; IWGSC, 2018). The SV density is much higher in the distal regions than in the proximal regions: 38 vs 8%, respectively (Figure 3). It also revealed the presence of one peak representing an increase of local PAV density in the central chromosomal region: 6 Mb at position 514–519 Mb, reflecting a single large region of CS absent in the genomes of East-Asian *T. aestivum*, *T. macha*, *T. carthlicum*, one *T. spelta* (*Tsp_Epeautre_noir_barbu_velu*), and one *T. dicoccum* (*Tdi_ 45309*). This region contains 18 genes of which a cluster of four duplicates (sharing >99% identity). This suggests a recent insertion of a DNA segment in the common ancestor of East-Asian *T. aestivum*.

### Phylogenetic Analysis

**FIGURE 4:**
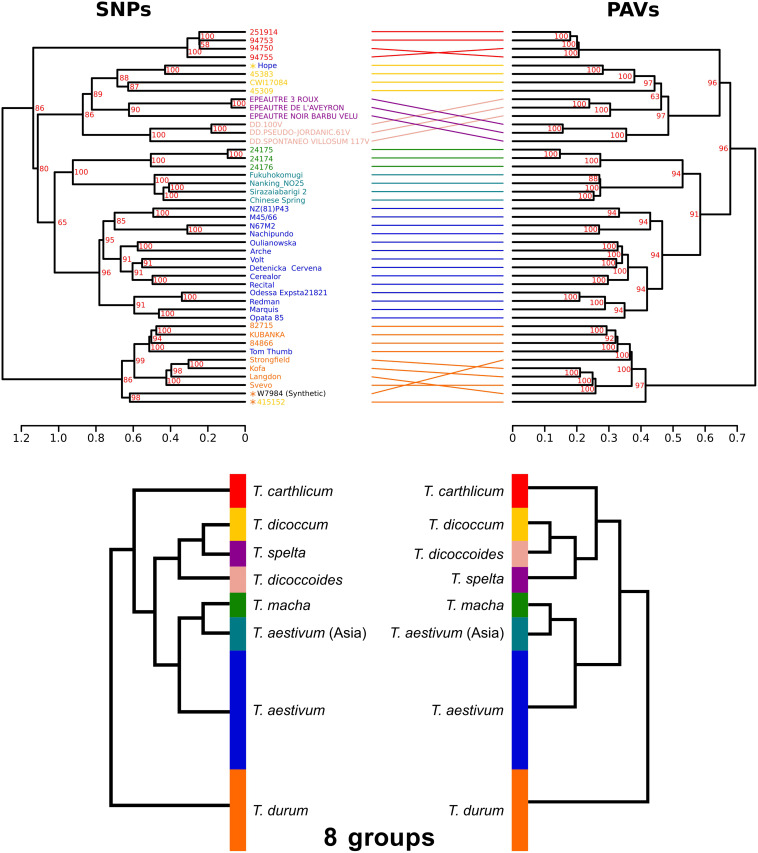
Dendrograms of the 45 *Triticum* accessions based on SNPs and PAVs. The two dendrograms represent the same eight monophyletic groups corresponding to the species classification: *T. aestivum* from Asia (light blue), other *T. aestivum* (blue), *T. spelta* (purple), *T. carthlicum* (bright red), *T. macha* (pale green), *T. durum* (orange), *T. dicoccum* (yellow), and *T. dicoccoides* (light red). Synthetic_W7984, *Tae_Hope, Tae_Tom-Thumb*, and *Tdi_*415152 are labeled with an asterisk indicating that their position in the tree does not fit the species classification. Bootstrap values are indicated in red. A simplified phylogeny is proposed on the bottom panel.

The phylogenetic relationships between the 45 accessions were investigated using either SNPs or PAVs. SNP-based and PAV-based dendrograms were mainly consistent, although some minor differences were observed (Baker’s gamma index = 0.97; Figure 4). Both dendrograms defined the same eight monophyletic groups corresponding to the seven *Triticum* species of our panel with *T. aestivum* split into two groups: East-Asian accessions (China and Japan) vs all other accessions, in agreement with a recent work on a large diversity panel of *T. aestivum* (Balfourier et al., 2019). The tree topology shows that the three hexaploid species originated from independent hybridization events and do not share a common tetraploid parent. Hexaploid *Triticum* do not form a monophyletic group: *T. macha* is branched with East-Asian *T. aestivum*, while *T. spelta* is branched with the tetraploids. We also identified four accessions that were classified in a species but for which the position in the tree suggested a recent hybrid origin (see Discussion): *Tae_Hope* was branched with *T. dicoccum*, *Tae_Tom Thumb* was related to *T. durum*, and Synthetic_w7984 and Tdi_415152 were also related to *T. durum.*

## Discussion

The complexity of the wheat genome makes it a challenge for molecular analyses, and it requires dedicated strategies to avoid misleading interpretations due to limitations of short read-based technologies in a highly redundant, polyploid, and large genome. Here, as a pilot study, we reduced the complexity through chromosome sorting, which allowed us to avoid multiple mapping of reads on homeologous gene copies, which share on average 97% nucleotide identity (Ramírez-González et al., 2018). Because of this complexity, most of previous studies focused on the gene space; i.e., 2% of the genome. Here, we obtained a global view of the variability at the whole chromosome scale, not only around genes but also for the 84% of TEs that shaped the chromosome. In addition, we selected a highly diverse panel of accessions, belonging to different species, with different ploidy levels. Indeed, it is well known that diversity within bread wheat is low because of the domestication and selection bottlenecks; thus, here we would like to get better insights into the extent of the potential reservoir of diversity present in the related species. We detected 1,179 variable genes (including CNV and PAV); i.e., 19% of the genes (or copies) sampled are not conserved among the seven *Triticeae* species. In addition, 330 genes not present in CS IWGSC RefSeq v1.0 were detected in several accessions. In total, variable genes account for 23% of chromosome 3B pangenome. In barley, a closely related *Triticeae* species, SVs were estimated to affect 15% of sequences and 9% of genes (Muñoz-Amatriaín et al., 2013), which is in the same range that what was found here. Thus, our results suggest that polyploidy did not trigger a significant boost in variability.

By comparing wheat to other grasses, we previously showed that distal chromosome regions evolve at a higher rate than the central region and concentrate most of the recently duplicated genes (Glover et al., 2015). Here, we demonstrate that this is also true at the intraspecific level for wheat, agreeing with several recent studies showing that large genomic variation mostly affects the distal regions (Rimbert et al., 2018; Balfourier et al., 2019; Gutierrez-Gonzalez et al., 2019). This genome partitioning was also the main outcome of the barley genome sequence analysis (Mascher et al., 2017), suggesting that this is a common feature to the *Triticeae* which share a genome architecture under constraint. This partitioning is associated with the higher frequency of meiotic recombination in the distal regions with fast-evolving regions being the regions where most of crossovers occur (Darrier et al., 2017). By building haplotypes, we confirmed the presence of a very large haplotype block of 111 Mb, which includes the centromere, showing that recombination did not break the genetic linkage in this region. In contrast, haplotype blocks are very small in the distal regions showing that the most variable regions are also the regions that recombined in the recent wheat history. This organization might have been selected for its efficiency to adapt to a changing environment. This is also in line with the findings that genes involved in the response to environment are the most variable. Our results on a diverse panel of *Triticum* accessions confirmed these observations on fewer accessions (Gutierrez-Gonzalez et al., 2019).

At the intraspecific level, for *T. aestivum*, we found 16% of dispensable/variable genes. Extrapolating this to the whole genome (i.e., 107,891 predicted genes), we can estimate a core genome of 90,628 genes and, on average, 17,263 variable genes. The difference with previously reported 64% (82,725) core genes predicted in Montenegro et al., 2017, may be explained by the use of higher depth of coverage and more stringent criteria to call an SV in our study.

If the diversity affecting genes has temptingly been estimated in previous pilot studies, the extent of variability of the repeat fraction of the genome was only poorly characterized. Here, at the scale of a complete chromosome, we show that the variability of the TE space is high: more than 60% of the ca. 200,000 elements shaping chromosome 3B of CS are either partially or totally absent in the related accessions. However, the number of new insertions detected either in CS or even in Asian *T. aestivum* (574 TEs) represents less than 1% of the TE content of the chromosome. Thus, it is obvious that TE transposition is not the main factor of this variability. This was confirmed by the strong conservation of the TE copy number estimated for each family. Indeed, only a few families are present in different proportions between accessions. This supports previous findings suggesting that the TE composition is highly controlled since A–B–D divergence, with no major TE burst (Wicker et al., 2018). Most of SVs affecting TEs originate from deletions, agreeing with previous conclusions made based on MITEs (Keidar-Friedman et al., 2018).

We used SV data to study the phylogenetic relationships between *Triticeae*. The tree topology suggests that hexaploid species originated from independent hybridization events and do not share a common tetraploid parent. Indeed, *T. macha* is branched with East-Asian *T. aestivum*, confirming previous hypotheses stating that *T. macha* originated from a hybrid cross between *T. aestivum* and wild emmer wheat (Matsuoka, 2011). Similarly, the position of *T. carthlicum* is consistent with previous findings indicating that it originated from a cross between domesticated emmer wheat and *T. aestivum* (Matsuoka, 2011). For *T. spelta*, it was proposed previously that it likely originated from an introgression of tetraploid wheat into hexaploid wheat (Blatter et al., 2004), which is in agreement with our phylogeny. Our study also revealed four cases where an accession classified in a species is more related to another species. *Tae_Hope* was branched with *T. dicoccum*; this can be explained by the fact that *Hope* has a recent parent which is an emmer wheat (INRA). Synthetic_w7984 is branched with *T. durum*, which is explained by the fact that it is a synthetic accession obtained by hybridization of a *T. durum* accession with *Ae. tauschii* (Sorrells et al., 2011). Tdi_415152, while being classified as a *T. dicoccum*, appeared to be a spontaneous hybrid between *T. dicoccum* and an unknown *T. durum*, explaining its clustering in the *T. durum* clade (INRA). Finally, *Tae_Tom Thumb* position among *T. durum* species suggests a similar scenario, with a potential hybrid origin from *T. aestivum* and *T. durum.* These results on multiple events leading to polyploid species provide information for future redefinition of species among the *Triticeae* and show the difficulty to trace the phylogenetic relationships between species in the *Triticum* clade due to recurrent hybridizations.

We established a strategy dedicated to complex genomes based on chromosome flow-sorting, Illumina resequencing, and fine-tuned bioinformatics approaches, to get highly accurate SVs affecting genes and the TE space at a wide scale of diversity. This study establishes the foundation for future pangenomic analyses at a larger scale and provides insights into the complex phylogenetic relationships between *Triticum* species.

## Materials and Methods

### Plant Material

We selected 45 *Triticum* accessions, including Chinese Spring, originated from 22 countries in 4 continents. Seeds were provided by the Biological Resource Center (CRB) of GDEC (INRAE-UCA, Clermont-Ferrand, France). This panel included three hexaploid and four tetraploid species: 19 *Triticum aestivum* (6×), three *Triticum macha* (6×), three *Triticum spelta* (6×), seven *Triticum durum* (4×), three *Triticum dicoccoides* (4×), four *Triticum dicoccum* (4×), and four *Triticum carthlicum* (4×) and one hexaploid synthetic accession (*Synthetic w7984*; 6×) (Table 1).

### Flow Sorting and Sequencing Chromosome 3B

Flow cytometry was used to sort chromosome 3B DNA of the 45 accessions following the protocol detailed in Vrána et al. (2000). Sorted chromosomal DNA was then amplified as previously described in Šimková et al. (2008) to get sufficient amount of DNA for sequencing. Each DNA sample was then sequenced with Illumina HISEQ-2000 using 2 × 100 bp paired-end reads (ENA project number PRJEB31708). For Chinese Spring, we used Illumina reads produced on flow sorted chromosome 3B DNA previously available [(Choulet et al., 2014); ENA project number PRJEB7360; Illumina runs ERR634934, ERR634939, ERR634942, ERR634936].

### Sequence Analyses

#### Read Mapping and Quality Assessment

Reads obtained for the 45 samples were mapped using BWA-MEM v0.7.12 (Li and Durbin, 2010) on the reference genome sequence of *T. aestivum* cv. Chinese Spring: IWGSC RefSeq v1.0 which includes the latest high-quality assembly of chromosome 3B pseudomolecule and the annotation of gene models IWGSC RefSeq Annotation v1.0 (IWGSC, 2018). We used samtools/1.3.1 (Li et al., 2009) to filter the alignments and bedtools/2.26 (Quinlan and Hall, 2010) to calculate the depth of coverage. A mapping quality (mapq) threshold of 11 (samtools view −q 11) was applied to filter out multi-mapped reads.

The coverage (i.e., proportion of chr3B covered by reads) and the depth of coverage (DoC) were calculated by considering the number of reads mapped with mapq ≧11 on the non-repeated fraction of the chromosome 3B (including genes, non-genic low-copy sequences, and ISBPs) accounting for 134 Mb. We used a DoC threshold of 5× to consider that a region was covered. To check for potential translocation between chromosomes 3B and 2B, we searched for reads mapped (mapq ≧11 and DoC > 5×) over at least 50 kb on chromosome 2B. To estimate the extent of local DoC variations across chromosome 3B, we used ISBPs conserved in all 45 accessions. First, we filtered out reads that were unmapped, not primary aligned, with supplementary alignments, and with quality <11 (samtools view −F 2308 −q11). Then, we discarded PCR duplicates (samtools rmdup). Finally, we counted reads that overlap TE insertion sites (i.e., unique k-mer) over at least 20 bps on each side of the junction using “bedtools coverage” (parameters: −f 0.6 −F0.9). To call SVs and SNPs, we filtered out reads using samtools view (−F 2308 −q11) and PCR duplicates using samtools rmdup.

#### SNP Calling

SNPs were called on non-repeat fraction of chr3B (134 Mb) using bam files generated at the previous step and with samtools mpileup (options: **−**C 50 **−**q 30 **−**Q 20) and Varscan2/2.3.9 (options: mpileup2snp –min-coverage 10 –min-reads2 10 –strand-filter 1 –min-freq-for-hom 0.85 –min-var-freq 0.02; Koboldt et al., 2012). For diversity analysis and haplotype assessment, we selected only SNPs of class 1 and 2; i.e., SNPs for which the 45 genotypes are homozygous (alleles AA or BB) or with the two homozygous and the potential heterozygous alleles (AA, AB, and BB). Then, haplotype blocks were computed using plink (parameters: –blocks no-pheno-req –allow-no-sex –blocks-max-kb 500000 –indep-pairwise 500000 5 0.99; v1.90b6.2; Purcell et al., 2007) on the previously generated VCF file. Based on the N50 of the haplotype block size, large haplotype blocks were considered as >3.1 Mb. To validate the presence of an upCNV, we searched for the presence of pseudo-heterozygous SNPs as evidence for the presence of several slightly divergent copies of the same gene, whose reads map one single gene in the reference. To discriminate pseudo-heterozygous SNPs from true residual heterozygosity, we considered only cases where all homozygous accessions share the same allele (no homozygous variant).

#### Phylogenetic Analyses

To get robust markers, we first pruned SNPs using plink (parameters: –blocks no-pheno-req –allow-no-sex –blocks-max-kb 500000 –indep-pairwise 500000 5 0.99; v1.90b6.2; Purcell et al., 2007) while keeping undetermined values (NA) in our genotyping matrix. We then used R package pvclust on both SNP and PAV matrix to perform a hierarchical clustering (function: pvclust(), distance: simple matching, clustering: Ward.D2, Bootstrap: 10000). Dendrograms were then compared and aligned using R package dendextend (Galili, 2015). Entanglement score estimated the quality of the dendrogram alignment, zero being perfectly untangle (SNP tree vs PAV tree = 0.01). To evaluate the similarity between the two dendrograms, Baker’s gamma index (Baker, 1974) was calculated using dendextend function cor_bakers_gamma. We then performed permutation test for the calculation of the statistical significance of Baker’s gamma index. For that, we compared (i) the SNP dendrogram to itself (Baker’s gamma index = 1, i.e., theoretical perfect case of identical trees comparison), (ii) SNP dendrogram to a dendrogram obtained after random shuffling of the SNP dendrogram (iteration = 100, mean Baker’s gamma index = 0.005), and (iii) SNP dendrogram to PAV dendrogram (Baker’s gamma index = 0.97).

#### Detection of CNVs and PAVs Affecting Genes

We used the High Confidence gene models predicted in the IWGSC RefSeq v1.0 Annotation as a reference gene annotation of chromosome 3B (IWGSC, 2018). We used samtools to analyze read mapping data in regard to gene positions. We used the available annotation of TEs and bedtools “complements” function in order to create a non-repeated chromosome 3B sequence of 134 Mb (including non-coding low-copy DNA, genes, and ISPBs). Reads mapped on the non-repeated fraction of chromosome 3B were then filtered using samtools view: reads unmapped, not primary aligned, with supplementary alignments, and mapped with quality under 11 were filtered out (samtools view **−F 2308 −**q11). PCR duplicates were also filtered out using samtools rmdup. Read depth and coverage were finally calculated using bedtools coverage. Forty-seven genes not covered after mapping CS resequencing dataset were filtered out, leaving 6,082 genes that could be analyzed.

Genes were considered absent (PAV) when covered over less than 10% of their length, while considering only coding exons. For the detection of CNVs affecting genes, we calculated a normalized ratio between the read count for each gene in accession X and the read count in Chinese Spring. First, we normalized the read counts based on the proportion of the length of the gene covered by reads. Second, for each accession, we normalized read counts based on the total number of reads mapped on all chromosome 3B genes. Finally, we computed a log2ratio of the normalized read counts for each gene between accession X and Chinese Spring. For each accession, a boxplot of the 6,082 gene log2ratios was computed and we used the R native function boxplot upper and lower whiskers as thresholds to identify potential genes with up- or downCNVs, i.e., DoC outliers/extreme values as CNVs are expected to be the outliers. Normalizations and statistics were computed using homemade Perl and R scripts. In addition, pseudo-heterozygous SNPs were used to detect the presence of upCNVs (multiple slightly different gene copies). They were obtained after the SNP calling using samtools mpileup and Varscan2 mpileup2snp (v2.3.9, options: –min-coverage 10 –min-reads2 10 –strand-filter 1 –min-freq-for-hom 0.85 –min-var-freq 0.02). We selected only SNPs of class three (see paragraph “SNP calling”) as evidence for upCNVs. For downCNVs, we added a detection of highly duplicated genes across the CS chromosome 3B in order to build a list of candidate genes that might be present in fewer numbers of copies in other accessions. For that, we clustered chromosome 3B protein sequences with CD-HIT (Li and Godzik, 2006) using the following parameters: −c 0.95 (i.e., 95% identity), −s 0.5 (i.e., 50% maximum gene size difference between the shortest and largest gene in the cluster), and −g 1 (more accurate mode).

#### Detection of TE inDels and CNVs

We developed a Perl script (getISBPfromBed.pl; available at^1^) in order to extract 150 bps encompassing the junction between every TE predicted across CS chromosome 3B and its insertion site, each corresponding to a potential 5′and 3′ ISBP marker. In case of major overlap between two neighbor ISBPs (>99 pbs), we kept only one ISBP to avoid redundancies. ISBPs containing one or more Ns were filtered out. In addition, we discarded non–unique ISBPs (detected by mapping ISBP sequences using BWA–mem). These later represent typical cases where a TE was predicted as two or more adjacent fragments, leading to design ISBPs that are fully included into an element and, thus, are repeated. TE inDels were assessed by mapping reads on the set of ISBPs while keeping only 100–bp reads fully mapped with at maximum 2 mismatches, meaning that the junction between the TE extremity of the insertion site is covered.

CNVs affecting TEs were assessed by comparing read depth for a given TE family in CS and in other accessions. We mapped reads with BWA–mem on IWGSC RefSeq v1.0, including TEs, and selected all best alignment(s) for each read. We normalized the read counts based on the length of the read mapped and based on multiple mapping; i.e., when a read mapped two TE copies, we counted 0.5 per copy. For each TE copy in each accession, we calculated a cumulative weighted read count and calculated a fold change compared with CS. Only TEs covered in CS for at least 50× were analyzed.

#### Detection of PAVs and CNVs Affecting the Non–coding Non–repeated Fraction of Chromosome 3B

We built a BED file as a template to call PAVs only in genes, non–coding low–copy DNA, and TE extremities (ISBPs). We retrieved the positions of low–copy regions (including genes) as the complement to the TE positions. These segments were split into 100–bp windows, while discarding all segments smaller than 51 bps. We then added the positions of the complete list of 150 bp ISBPs to the BED file. Altogether, these regions account for 134 Mb split into 1,593,666 loci of 100/150 bps. Presence/absence of each locus was computed by analyzing read mapping data as described above. This BED file was also used to detect deletion blocks. Blocks were defined using a sliding window of 50 neighbor loci (with step = 5). Large deletion blocks were considered when at least two neighbor windows with more than 50% loci are absent. Distribution of SVs across the chromosome was analyzed in a sliding window of 10 Mb (step = 1 Mb), and segmentation analyses were computed with R package changepoint (penalty: “BIC,” penalty value: 0.05, method: “SegNeigh;” Chen and Gupta, 2012).

#### Assembly of Unmapped Reads and Gene Prediction

We used samtools to select unmapped reads (−F4) and to create 2 paired–end and 1 single end fastq files (−f 64, −f 128). We assembled these reads independently for each accession using MIRA (parameters: genome, *de novo*, accurate; v4.0.2, Chevreux, 2004) and kept only contigs larger than 500 bps. Potential contaminations were searched by aligning all contigs against the NCBI nt database (August 2018^2^) using BLASTn (parameters: −F F −e 1e-5, Altschul, 1997). Contigs with best hits outside the plant kingdom (id ≧ 80%, length overlap cutoff ≧200 bps) were considered contamination. Then, we searched for the presence of coding genes using Exonerate (options –maxintron 20000 –showtargetgff TRUE –model protein2genome; v2.2.0 Slater and Birney, 2005) and the predicted proteins originating from 9 plant genomes: *Sorghum bicolor*, *Zea mays*, *Oryza sativa*, *Brachypodium distachyon*, *Aegilops tauschii*, *Hordeum vulgare*, *Triticum urartu*, *Populus trichocarpa*, and *Arabidopsis thaliana*. We finally clustered the gene models assembled in different samples using CD-HIT (parameters: −identity −c 0.95, coverage: −s 0.8 −g 1; v4.7). We kept only genes assembled for at least two accessions and not present in CS as new gene candidates.

## Data Availability Statement

The datasets presented in this study can be found in online repositories. The names of the repository/repositories and accession number(s) can be found below: https://www.ebi.ac.uk/ena, PRJEB31708.

## Author Contributions

RD, FCh, and EP conceived and designed the study. FCh coordinated the study. RD developed SV detection methods and performed SV discovery analyses. HR helped in the data curation. FB provided biological resources and helped to design the accession panel. JV and JD flow-sorted chromosome 3B, established the purity in sorted fractions, and prepared the amplified DNA. FCa sequenced DNA amplified from flow-sorted chromosome 3B. RD, FCh, and EP wrote the manuscript. JK contributed figures to the manuscript. HR, FB, JK, ED, JV, JD, and FCa provided comments and corrections on the manuscript. All authors read and approved the final manuscript.

## Conflict of Interest

The authors declare that the research was conducted in the absence of any commercial or financial relationships that could be construed as a potential conflict of interest.

## Footnotes

1 https://github.com/fchoulet/biotools

2 ftp://ftp.ncbi.nih.gov/blast/db/FASTA
