# Supplementary material for: Structural Variations Affecting Genes and Transposable Elements of Chromosome 3B in Wheats
Source: Front Genet. 2020 Aug 18;11:891. doi: 10.3389/fgene.2020.00891 (PMC7461782; doi:10.3389/fgene.2020.00891)
Supplement: Supplementary file 1 [file Data_Sheet_1.PDF]

Supplementary Figures and Tables

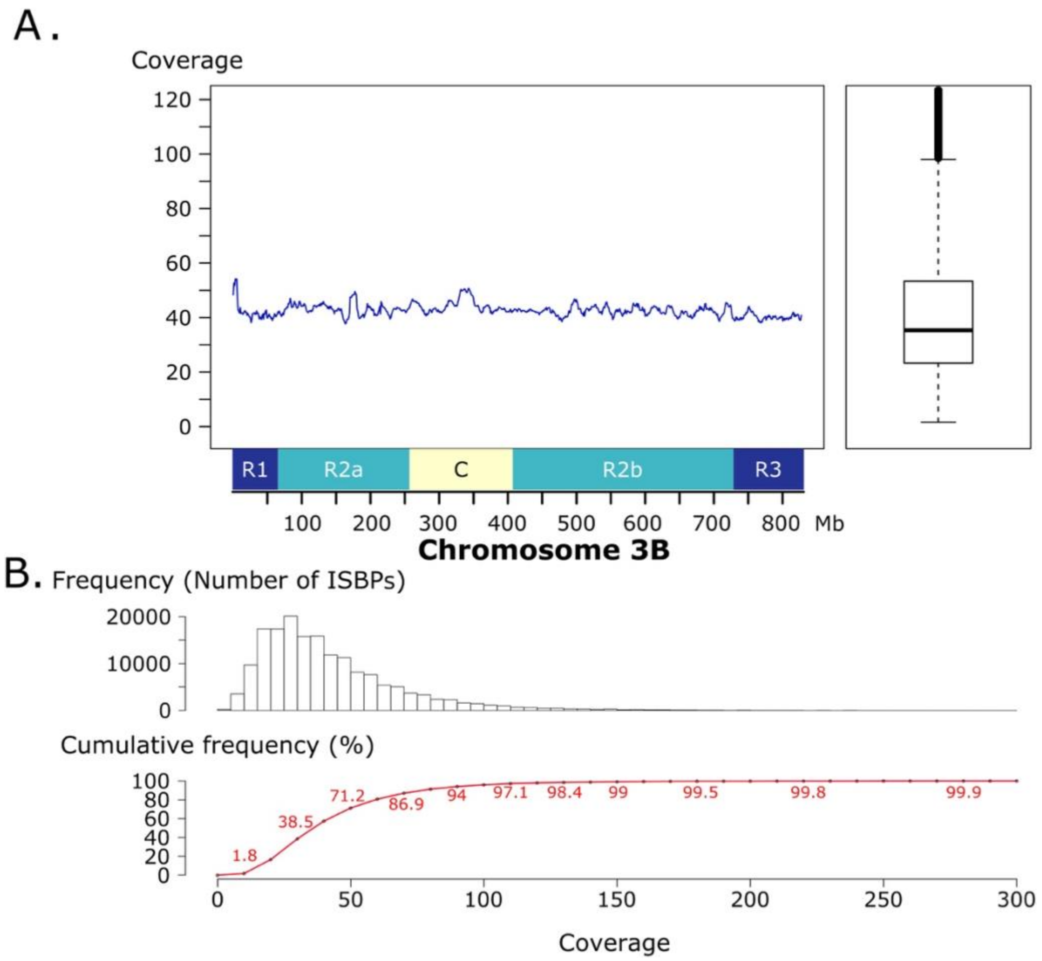

Figure S1: Variations of the depth of coverage (DoC) across chromosome 3B in CS estimated using mapping data over 170,906 ISBPs.
ISBPs are unique kmers (150 bps) at the genome-wide scale and, thus, are good candidates to evaluate DoC variations across chr3B. **A.** Distribution of DoC across chr3B for Chinese Spring (blue line) calculated in a sliding window of 10 Mb (step: 1 Mb). On the right is represented a boxplot of the DoC of all 10 Mb windows. **B.** Histogram and cumulative frequency plot (in red) of the DoC per window.

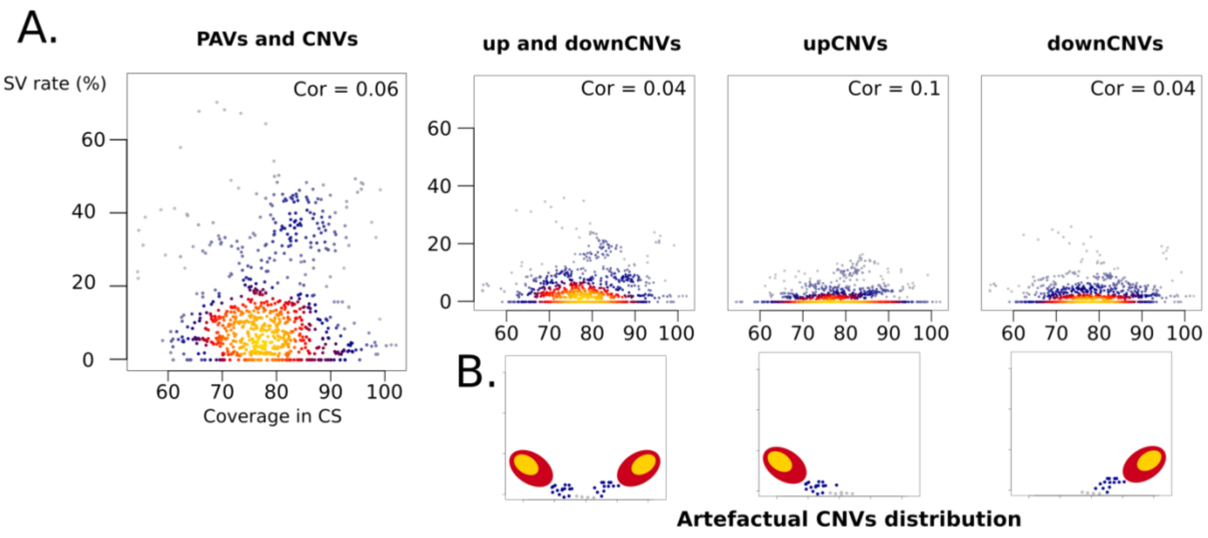

Figure S2: Local variations of DoC in CS and correlation with the number of SVs
A. Scatter plots representing the absence of correlation between the rate of SVs detected in 10 Mb windows and the DoC in CS. **B.** Schematic scatter plots representing the expected distribution if the detection of SVs would be biased by the DoC in CS. No correlation was detected between SVs and the variation of the DoC in CS.

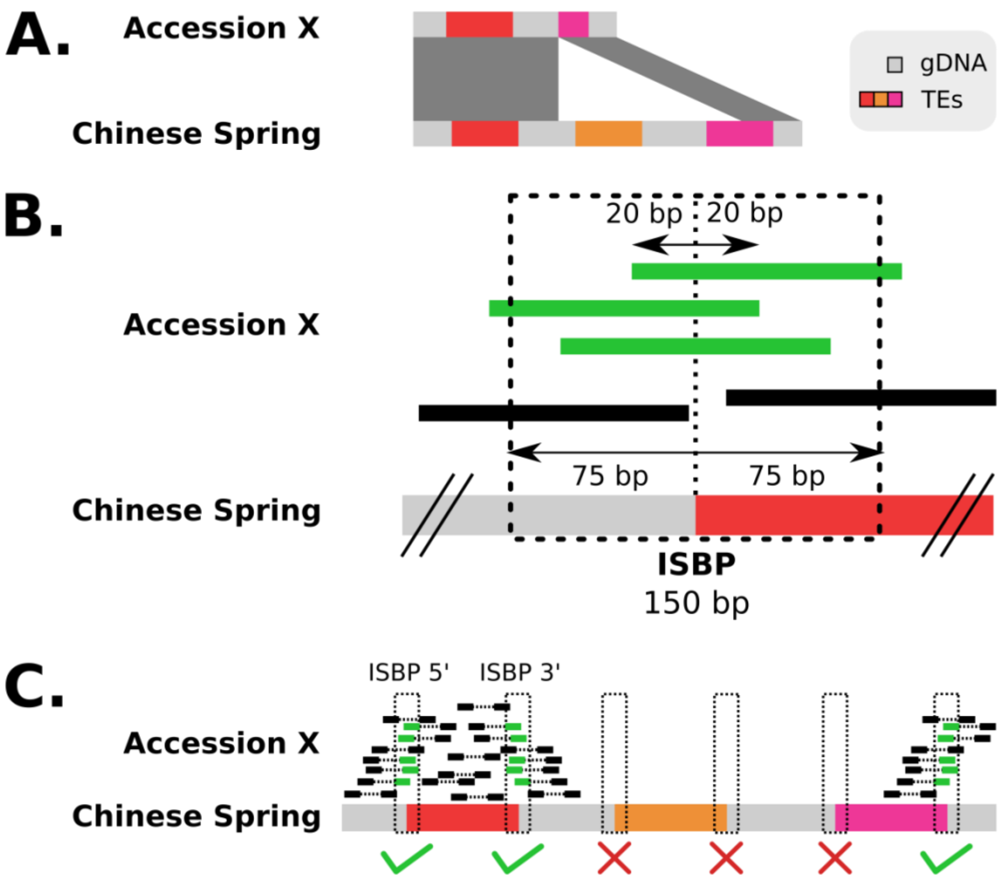

Figure S3: Strategy developed for the identification of PAVs affecting TEs based on read mapping on ISBPs
150 bps encompassing the extremity of a TE and its insertion site (75 bp on each side) was extracted at both the 5' and 3' ends of each TE, representing 5' and 3' ISBPs. An ISBP is a unique kmer at the genome-wide scale, contrary to the internal part of TEs. **A.** Example of an orthologous region between the reference CS and a query accession X. The region carries three TEs in the reference but only the red TE is conserved in X whereas the orange element is fully absent and the pink element is partially deleted. **B.** 101-bp reads were mapped on the 150-bp ISBPs and we considered only reads that mapped over more than 95 bps (represented in green, meaning at least 75+20 bps on each side of the junction *versus* reads represented in black that were filtered out). The presence of mapped reads indicates no TE InDel while absence of mapped reads is an evidence for InDel affected the ISBP. **C.** Presence of the red TE is revealed by reads mapped on both 5' and 3' ISBPs; complete TE-Indel of the orange element is revealed by the absence of mapped reads on both 5' and 3' ISBPs; partial TE-InDel of the pink element is revealed by reads mapped only on the 3' ISBP.

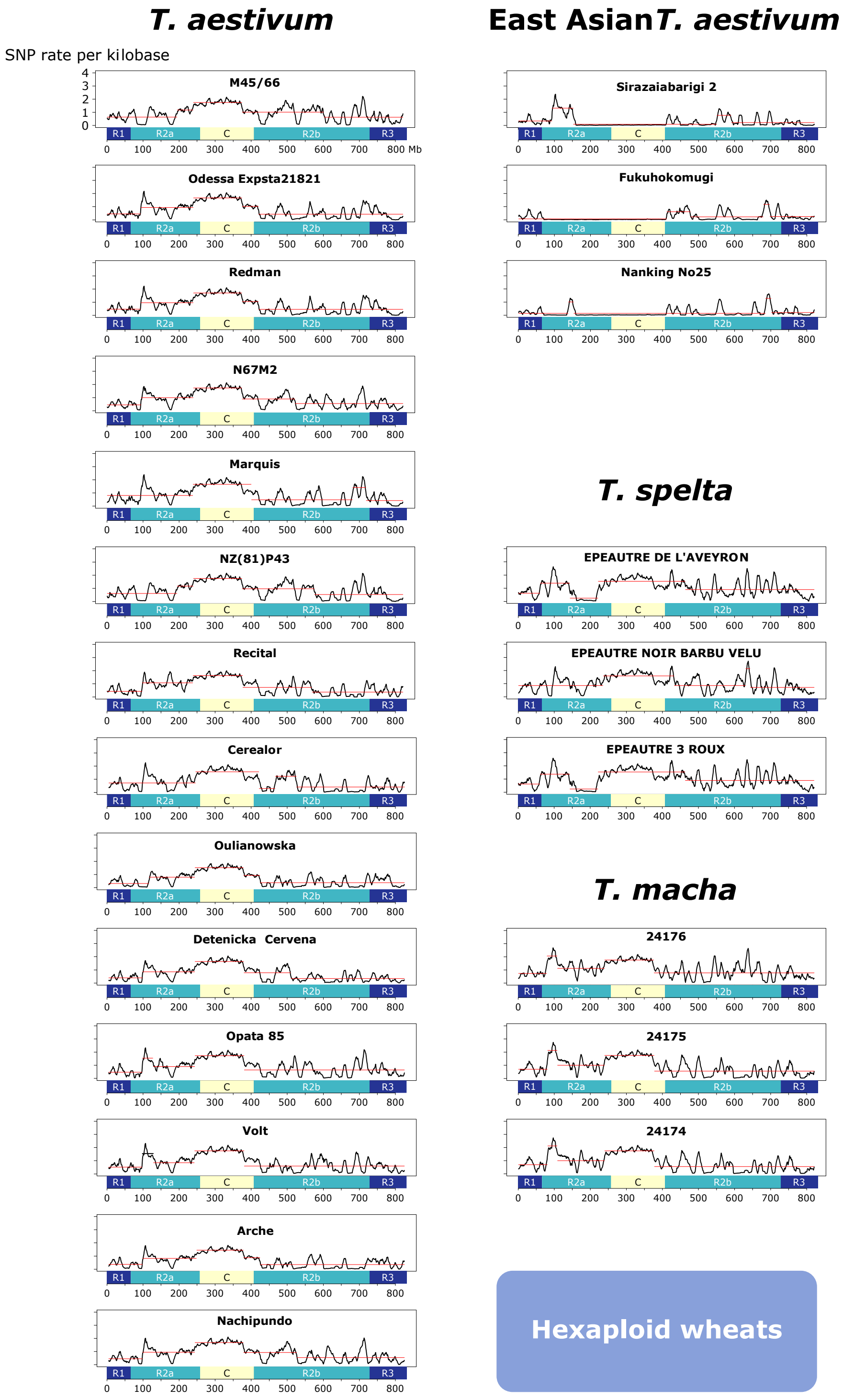

Figure S4: SNP density across chromosome 3B for the 23 hexaploid accessions

SNP density corresponds to the number of SNPs per kb calculated in a sliding window of 10 Mb (step=1 Mb). Segmentation was computed with R package Changepoint (penalty: BIC, value: 0.05, method: SegNeigh) and segments are represented by red lines.

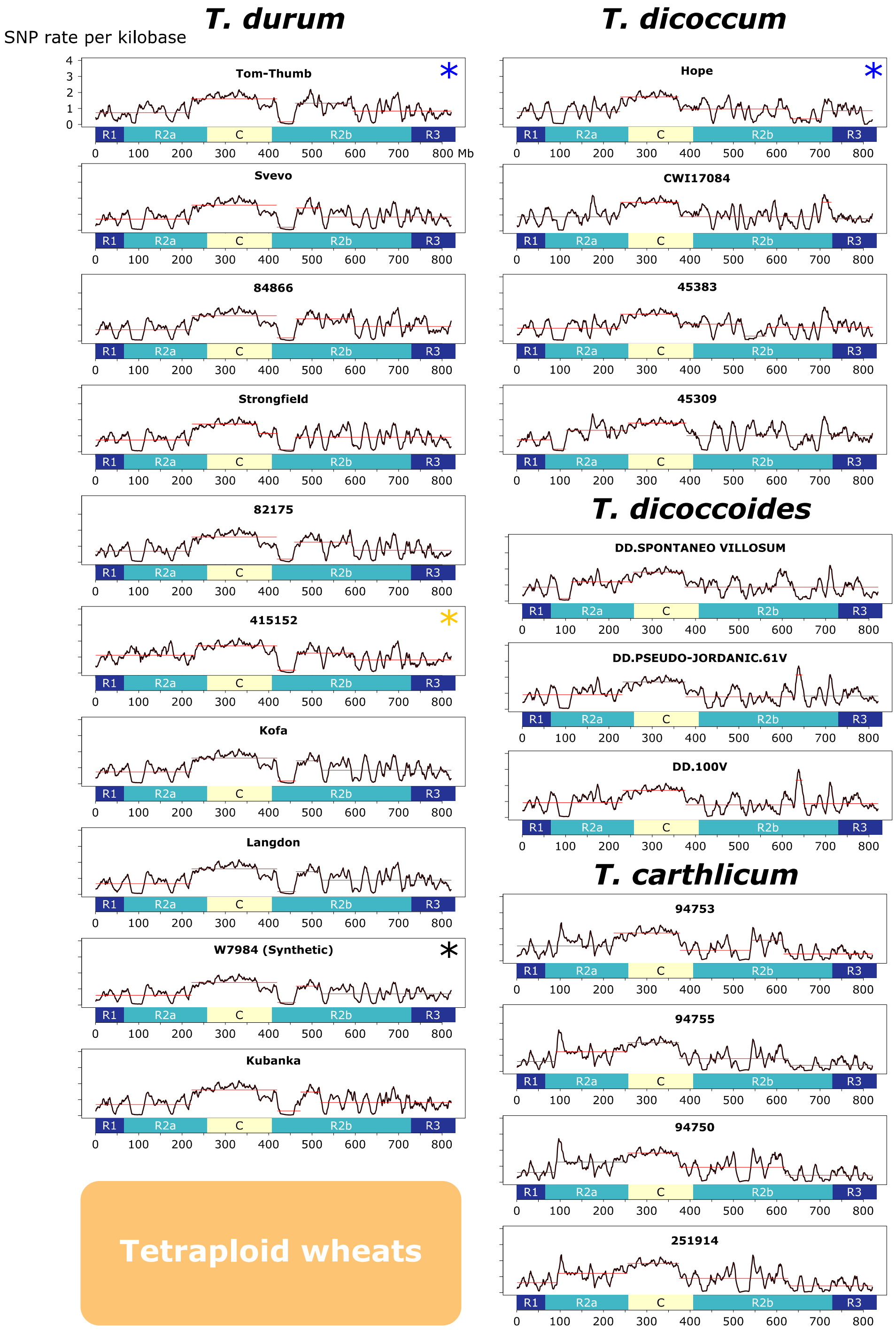

Figure S5: SNP density across chromosome 3B for the 21 tetraploid accessions

SNP density corresponds to the number of SNPs per kb calculated in a sliding window of 10 Mb (step=1 Mb). Segmentation was computed with R package Changepoint (penalty: BIC, value: 0.05, method: SegNeigh) and segments are represented by red lines

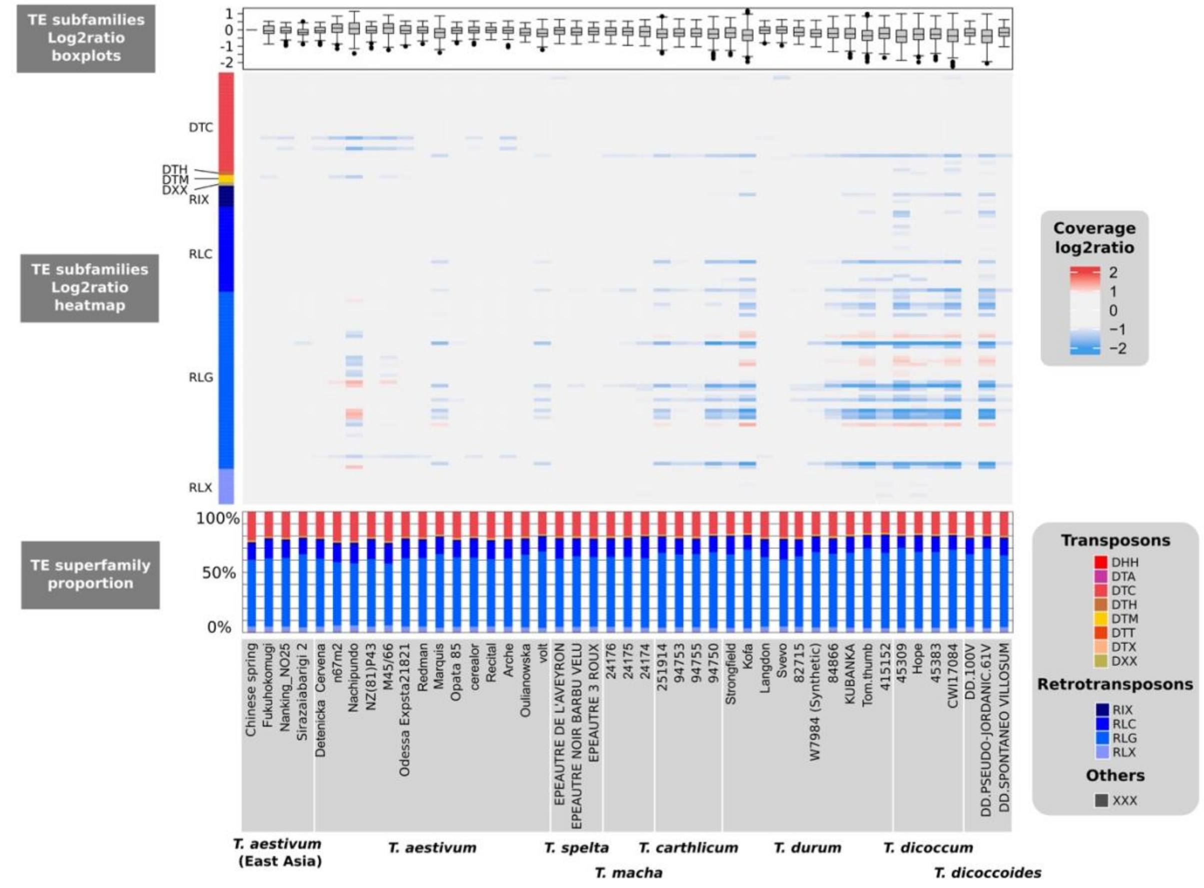

Figure S6: Variations of TE copy number per family on chr3B for 45 *Triticum* accessions

The abundance of each TE family (and subfamily) was estimated using DoC relatively to the abundance observed in the reference CS chr3B (expressed as log2ratio). Top panel: boxplots of the DoC log2ratios for all accessions which names are described at the bottom of the figure. Middle panel: heatmap of the relative abundance of all TE subfamilies compared to CS. Each line represents one TE subfamily. Positive and negative log2ratios, indicating higher and lower abundance in comparison to CS are represented in red and blue, respectively. Only subfamilies with a cumulative size of at least 1 kb were considered here. Bottom panel: barplots of the composition in TEs superfamilies for the 45 accessions. RIX: LINEs, RLG: Gypsy, RLC: Copia, RLX: unclassified LTR-retrotransposons, DHH: helitrons, DTA: hAT transposons, DTC: CACTA, DTH: Harbinger, DTM: Mutator, DTT: Mariner, DTX: unclassified transposons with TIRs; DXX: unclassified transposons.

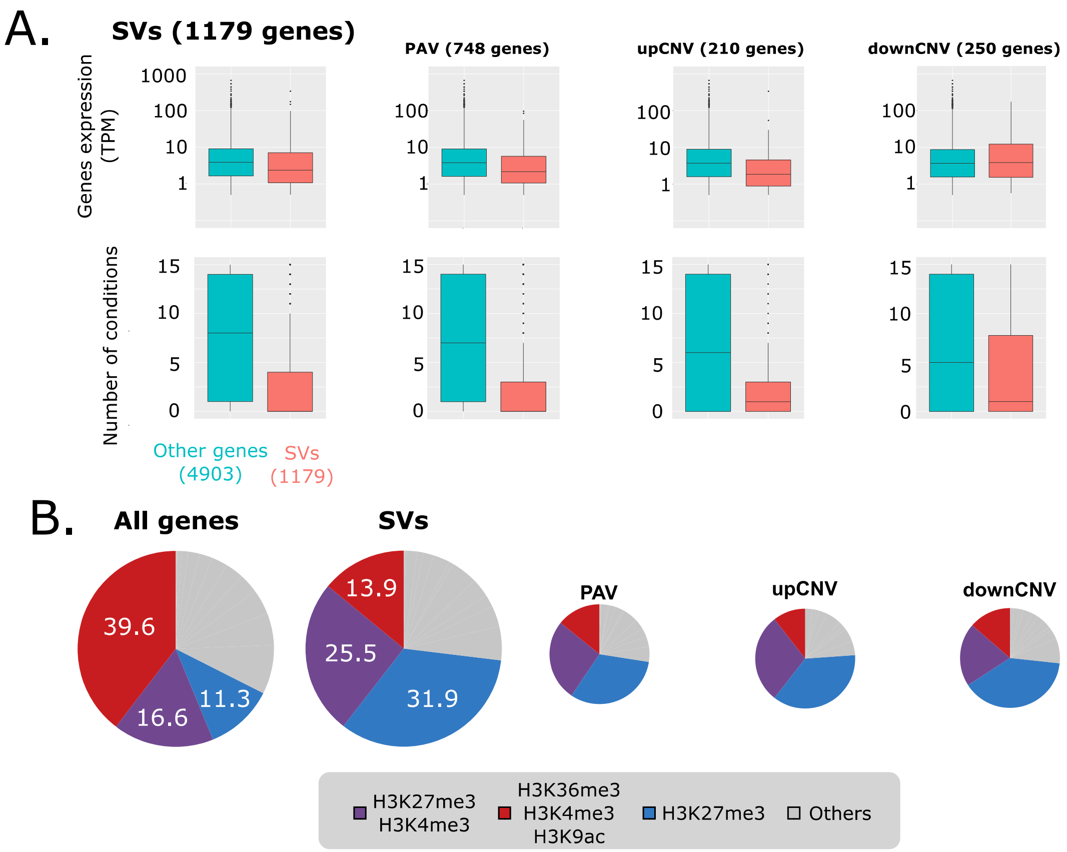

Figure S7: Expression levels and epigenetic marks associated with genes affected by SVs.

A. Boxplots of gene expression levels (in TPM; log10 scale) and expression breadths (in number of conditions out of 15 tested) for genes affected by SVs (red) and all other genes (cyan). B. Proportions of genes associated with different combinations of histone marks. The two left pie charts represent these proportions for all genes and genes affected by SVs. The three smaller pie charts on the right show details per type of SVs.

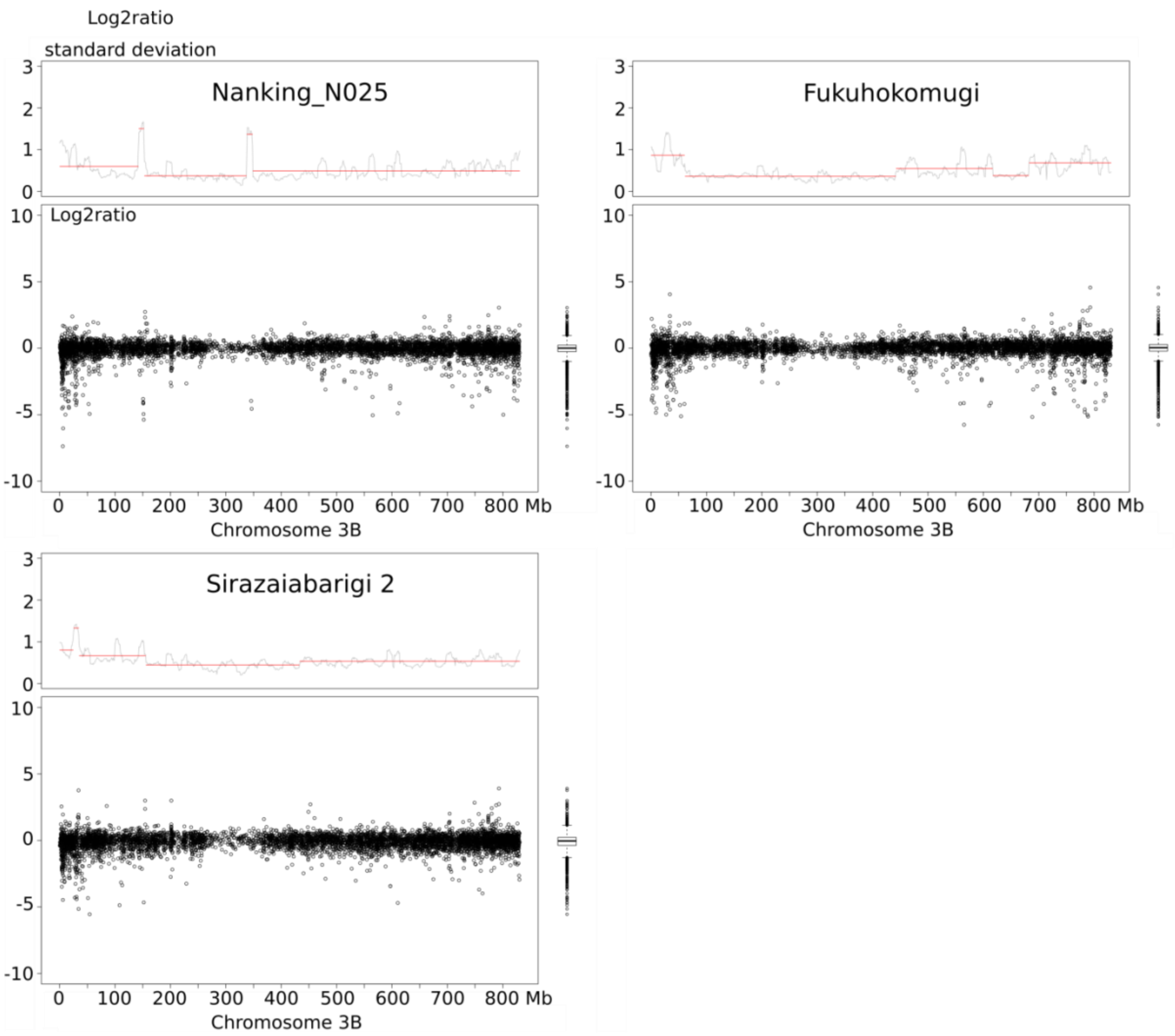

Figure S8: DoC log2ratios for East-Asian *T. aestivum* accessions

DoC log2ratios of all genes across chromosome 3B for 3 East-Asian *T. aestivum* accessions and standard deviation calculated in a 10 Mb sliding window (step=1 Mb, R package changepoint, penalty: "BIC", penalty value: 0.05, method: “SegNeigh"). A boxplot of the DoC log2ratios with upper and lower whiskers considered as thresholds to define up and downCNVs is represented on the right.

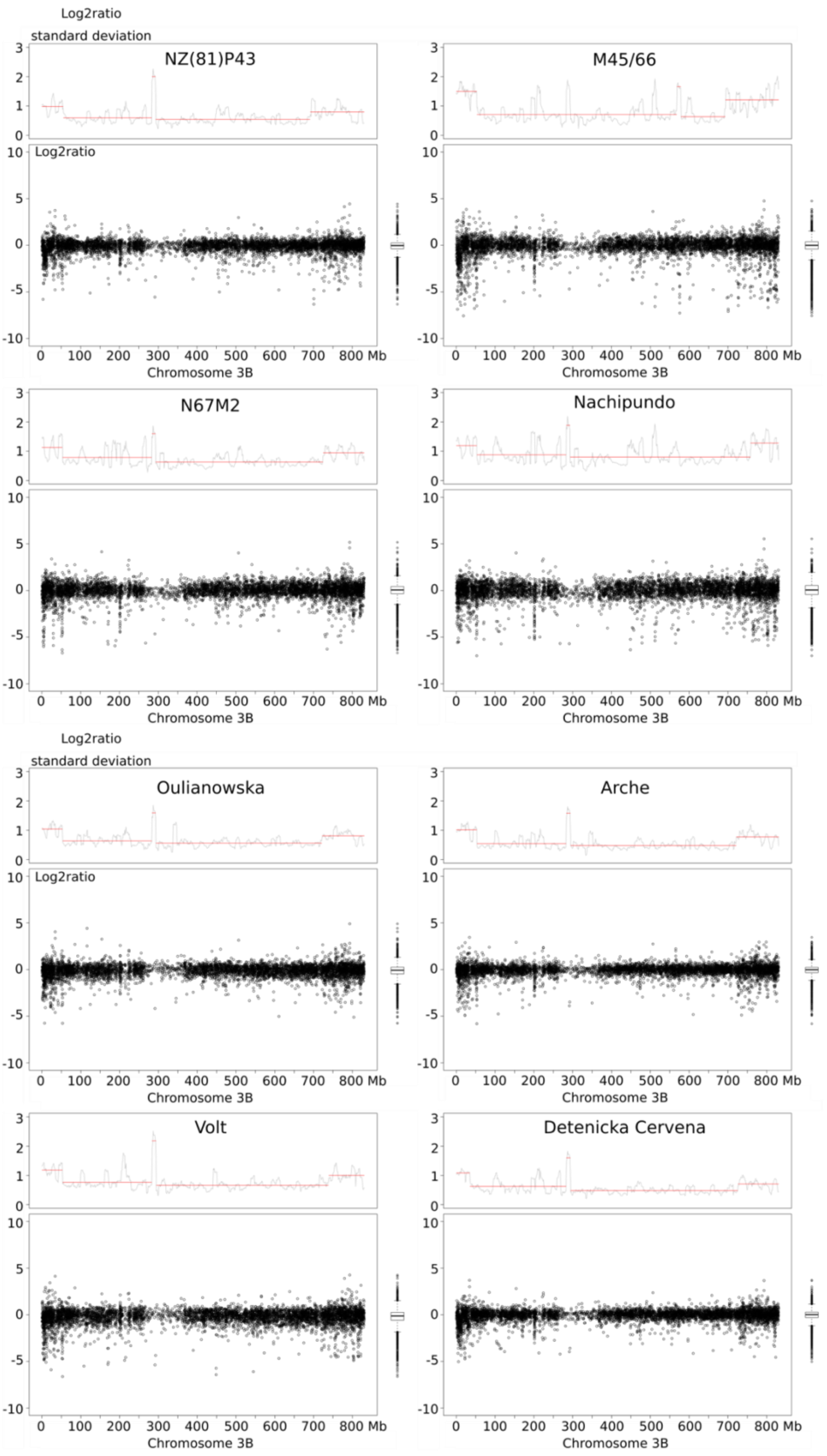

Log2ratio
standard deviation
NZ(81)P43
M45/66
N67M2
Nachipundo
Oulianowska
Arche
Volt
Detenicka Cervena
Log2ratio
0 100 200 300 400 500 600 700 800 Mb
Chromosome 3B

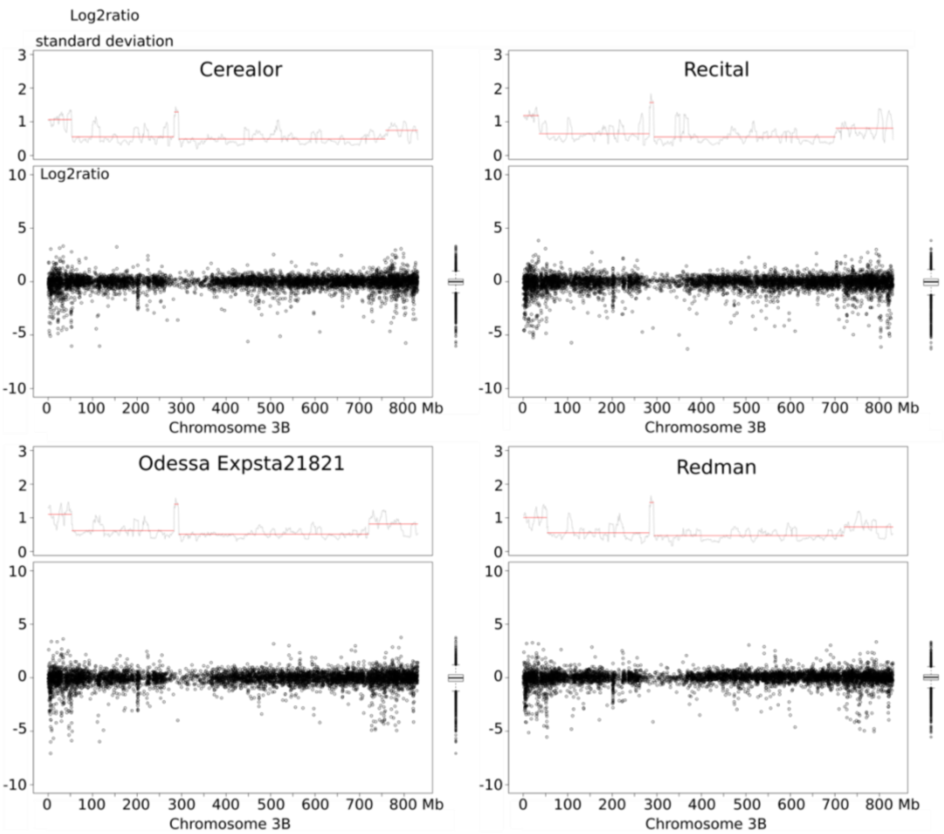
Log2ratio
standard deviation
Cerealor
Recital
Odessa Expsta21821
Redman
Log2ratio
3
2
1
0
10
5
0
-5
-10
0 100 200 300 400 500 600 700 800 Mb
Chromosome 3B

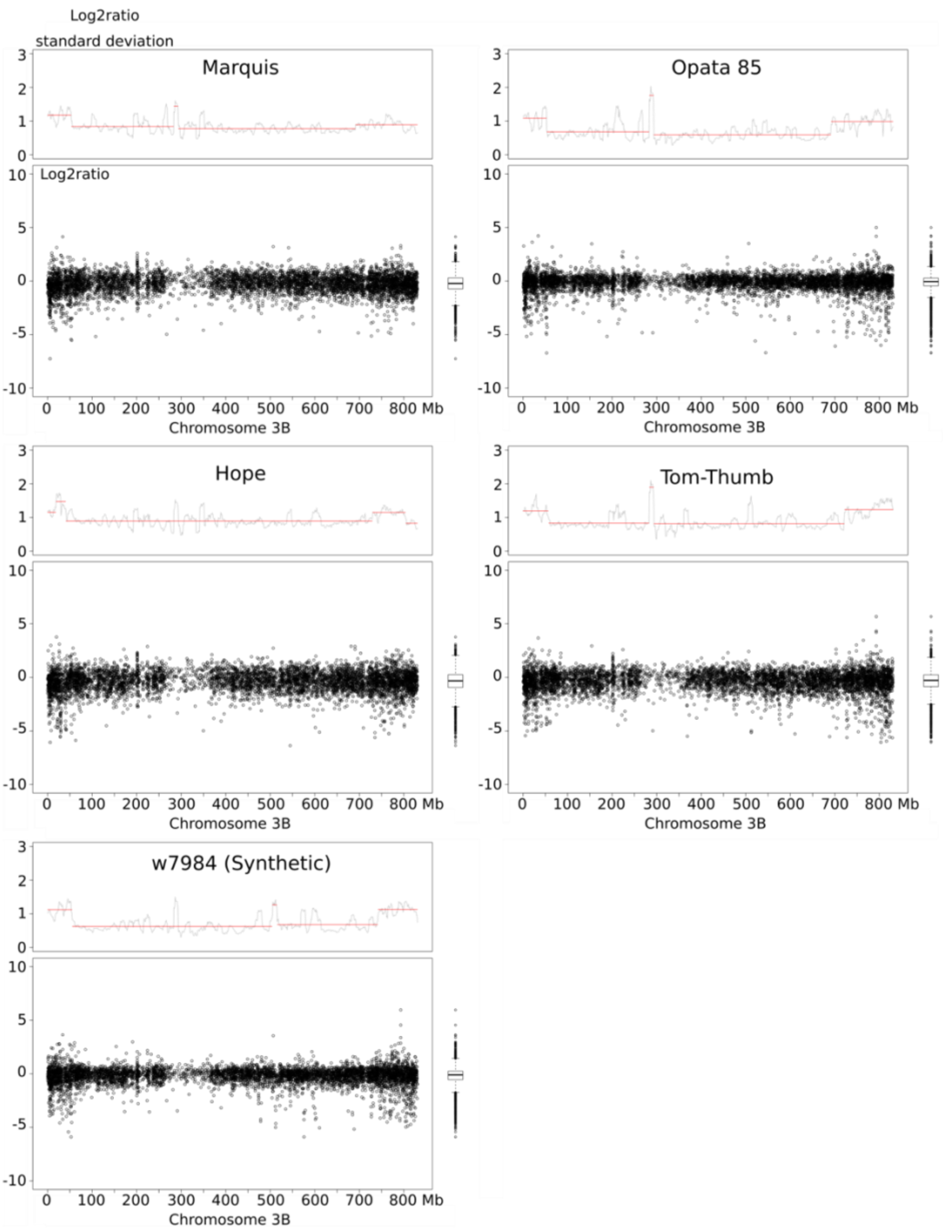

Figure S9: DoC log2ratios for *T. aestivum* accessions (excluding Asian accessions)
DoC log2ratios of all genes across chromosome 3B and standard deviation calculated in a 10 Mb sliding window (step=1 Mb, R package changepoint, penalty: "BIC", penalty value: 0.05, method: “SegNeigh"). A boxplot of the DoC log2ratios with upper and lower whiskers considered as thresholds to define up and downCNVs is represented on the right.

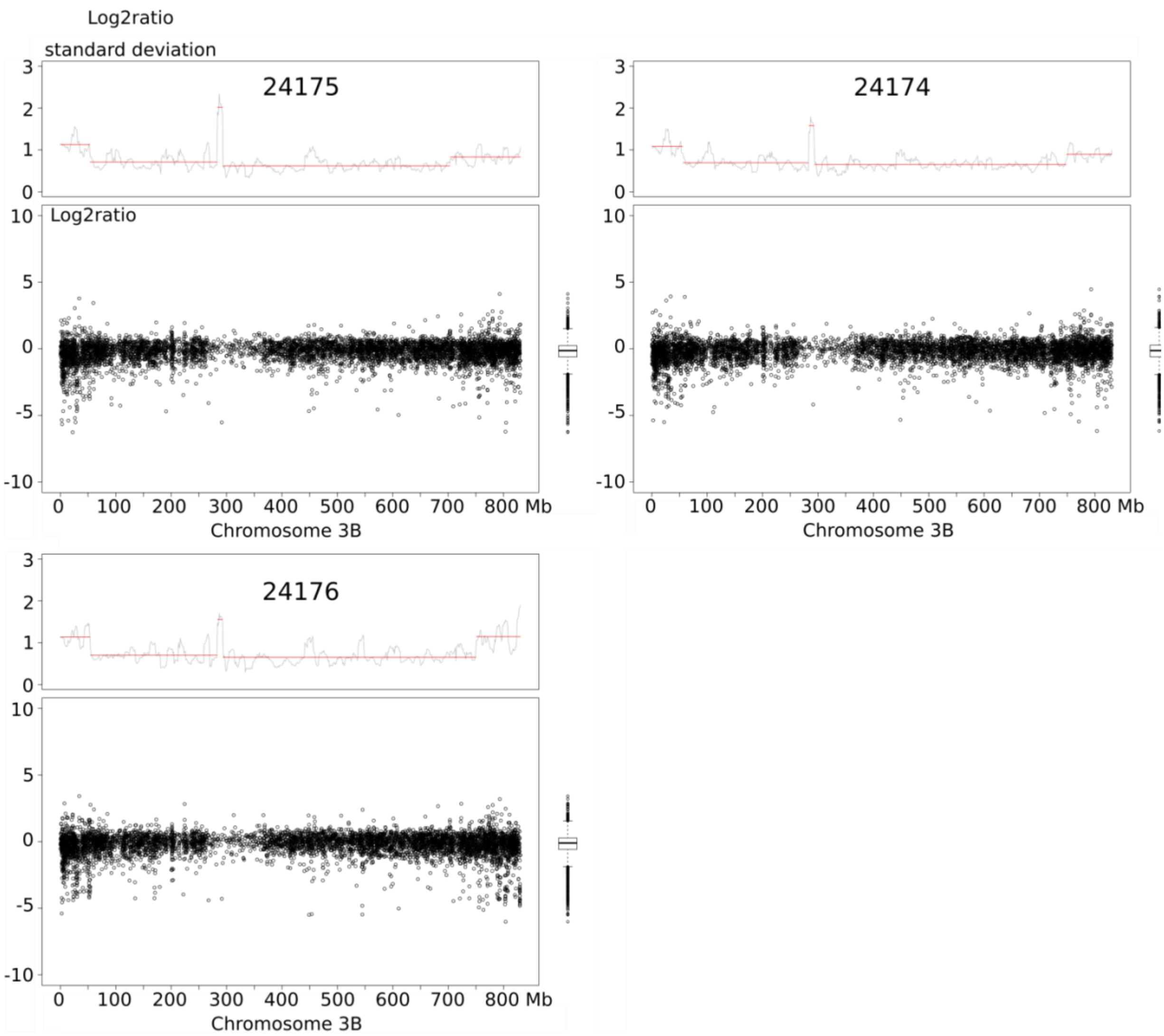

Figure S10: DoC log2ratios for *T. macha* accessions.

DoC log2ratios of all genes across chromosome 3B and standard deviation calculated in a 10 Mb sliding window (step=1 Mb, R package changepoint, penalty: "BIC", penalty value: 0.05, method: “SegNeigh"). A boxplot of the DoC log2ratios with upper and lower whiskers considered as thresholds to define up and downCNVs is represented on the right.

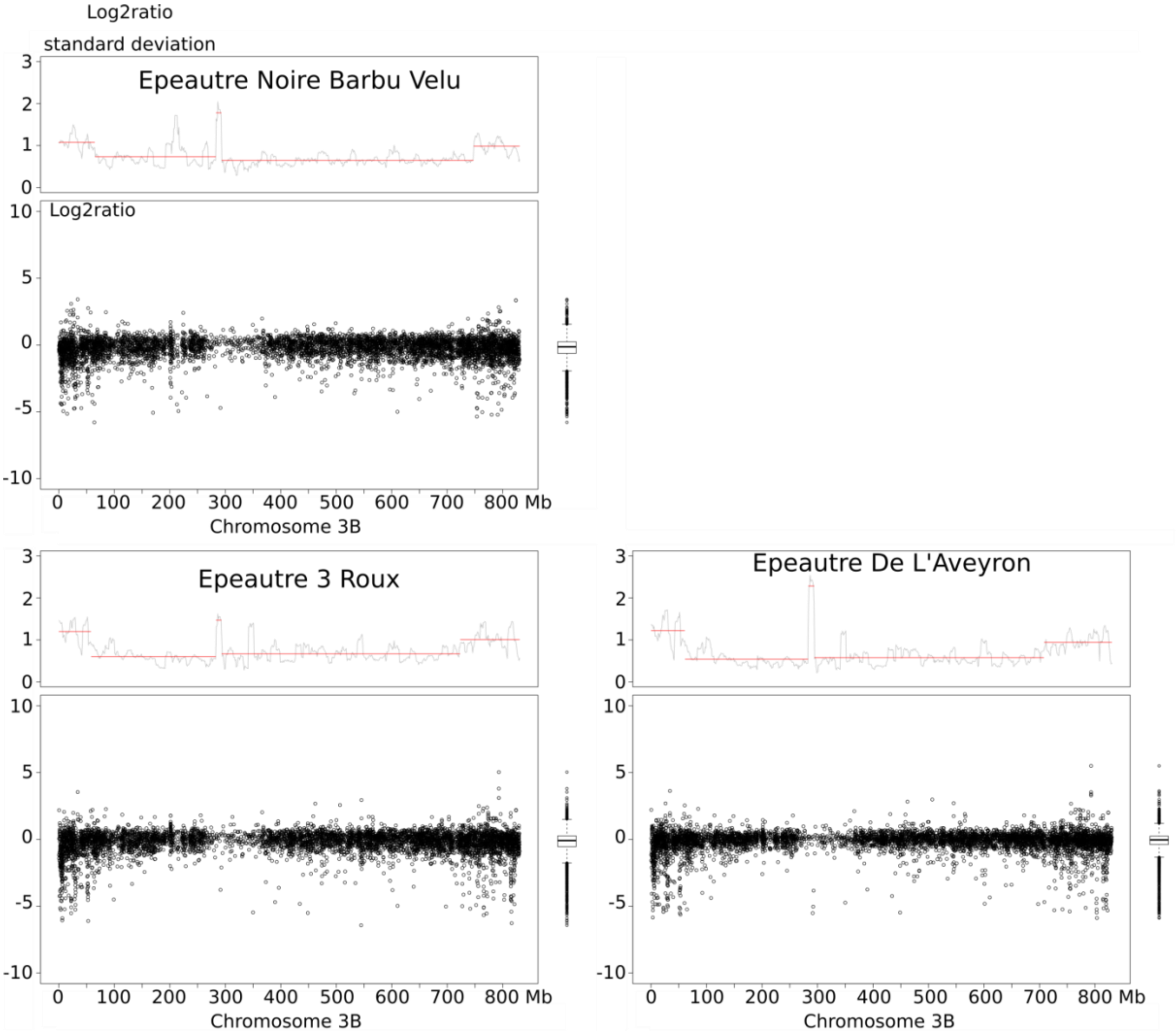

Figure S11: DoC log2ratios for *T. spelta* accessions.

DoC log2ratios of all genes across chromosome 3B and standard deviation calculated in a 10 Mb sliding window (step=1 Mb, R package changepoint, penalty: "BIC", penalty value: 0.05, method: “SegNeigh"). A boxplot of the DoC log2ratios with upper and lower whiskers considered as thresholds to define up and downCNVs is represented on the right.

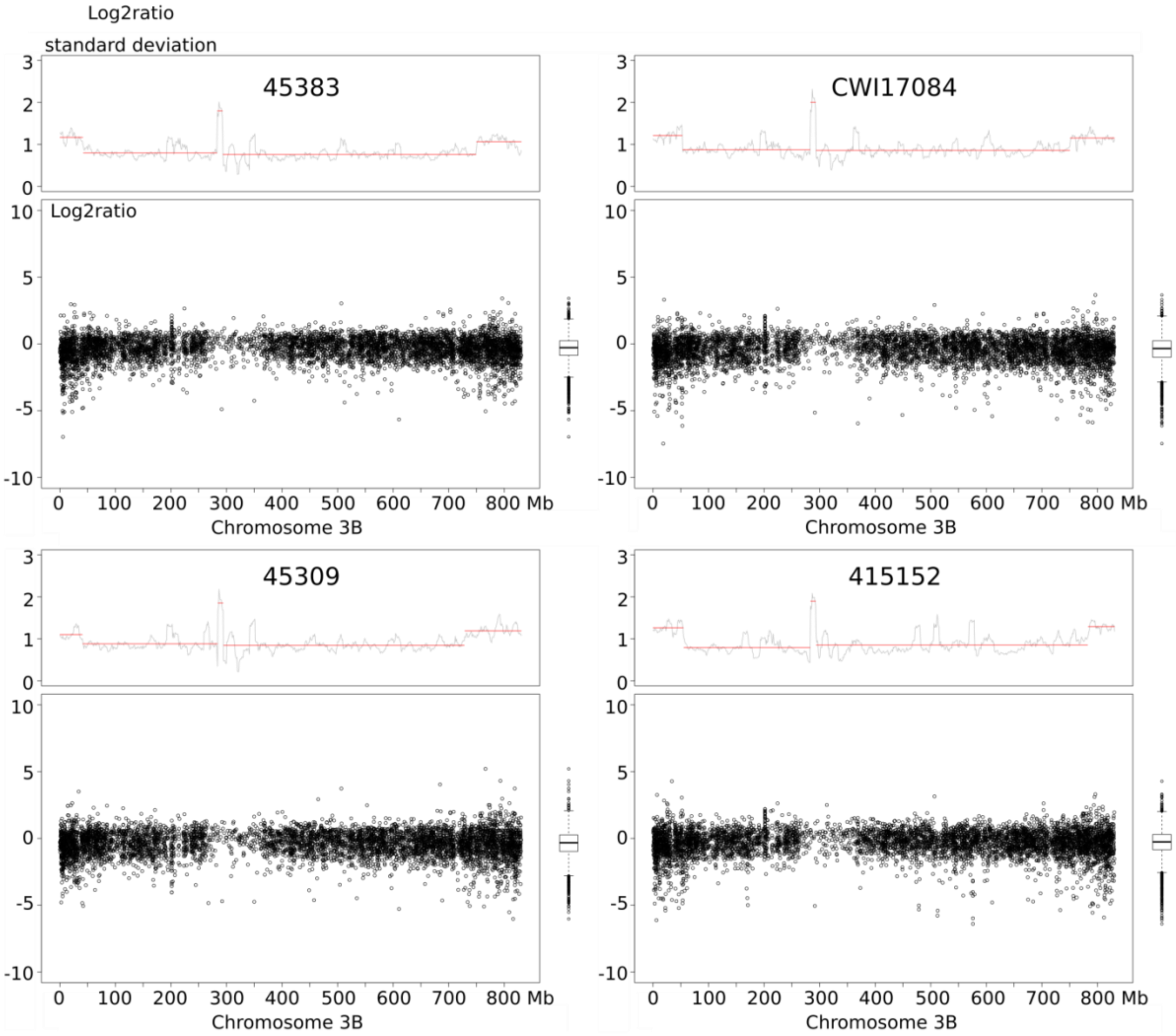

Figure S12: DoC log2ratios for *T. dicoccum* accessions.
DoC log2ratios of all genes across chromosome 3B and standard deviation calculated in a 10 Mb sliding window (step=1 Mb, R package changepoint, penalty: "BIC", penalty value: 0.05, method: “SegNeigh"). A boxplot of the DoC log2ratios with upper and lower whiskers considered as thresholds to define up and downCNVs is represented on the right.

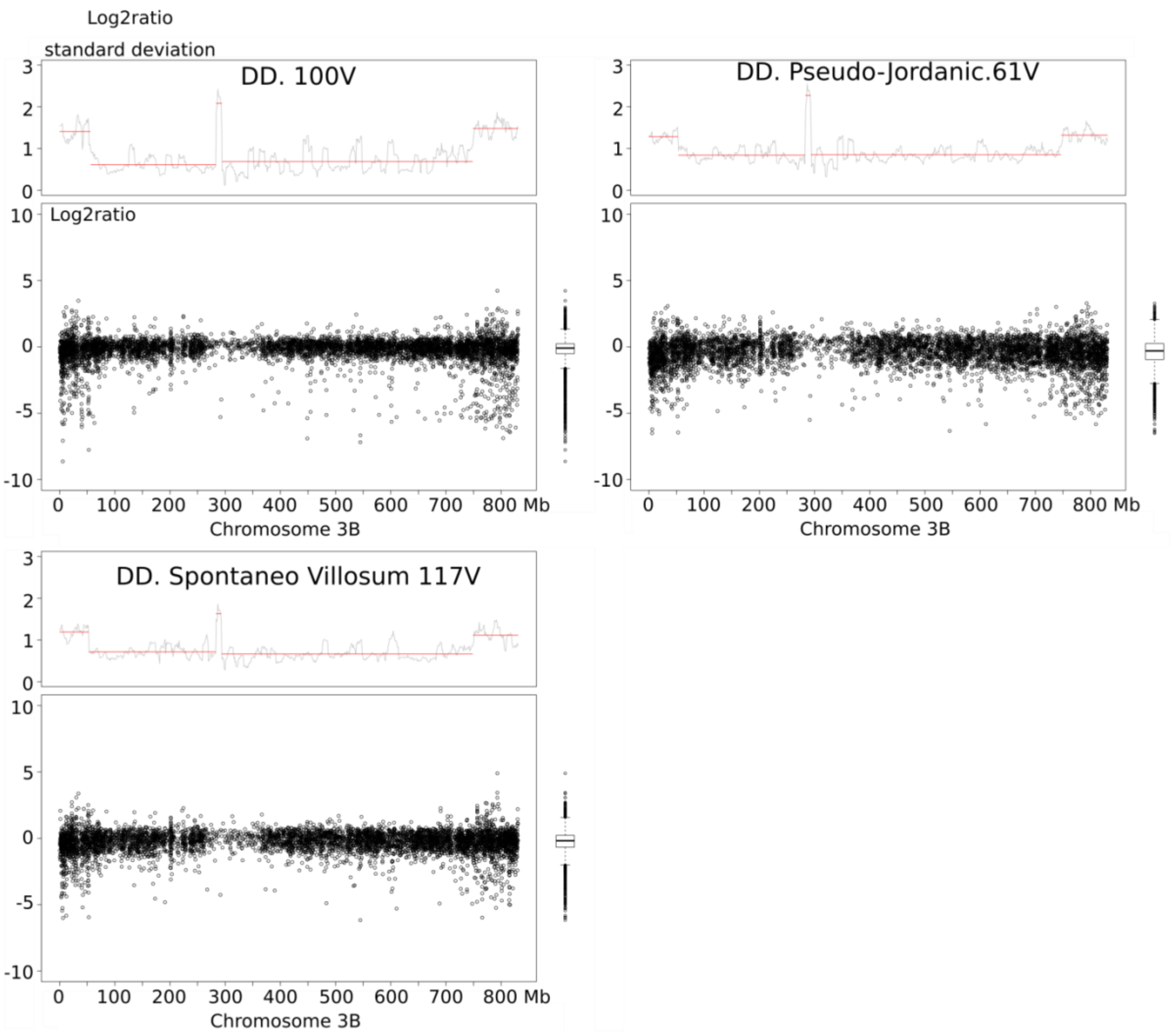

Figure S13: DoC log2ratios for *T. dicoccoides* accessions.

DoC log2ratios of all genes across chromosome 3B and standard deviation calculated in a 10 Mb sliding window (step=1 Mb, R package changepoint, penalty: "BIC", penalty value: 0.05, method: “SegNeigh"). A boxplot of the DoC log2ratios with upper and lower whiskers considered as thresholds to define up and downCNVs is represented on the right.

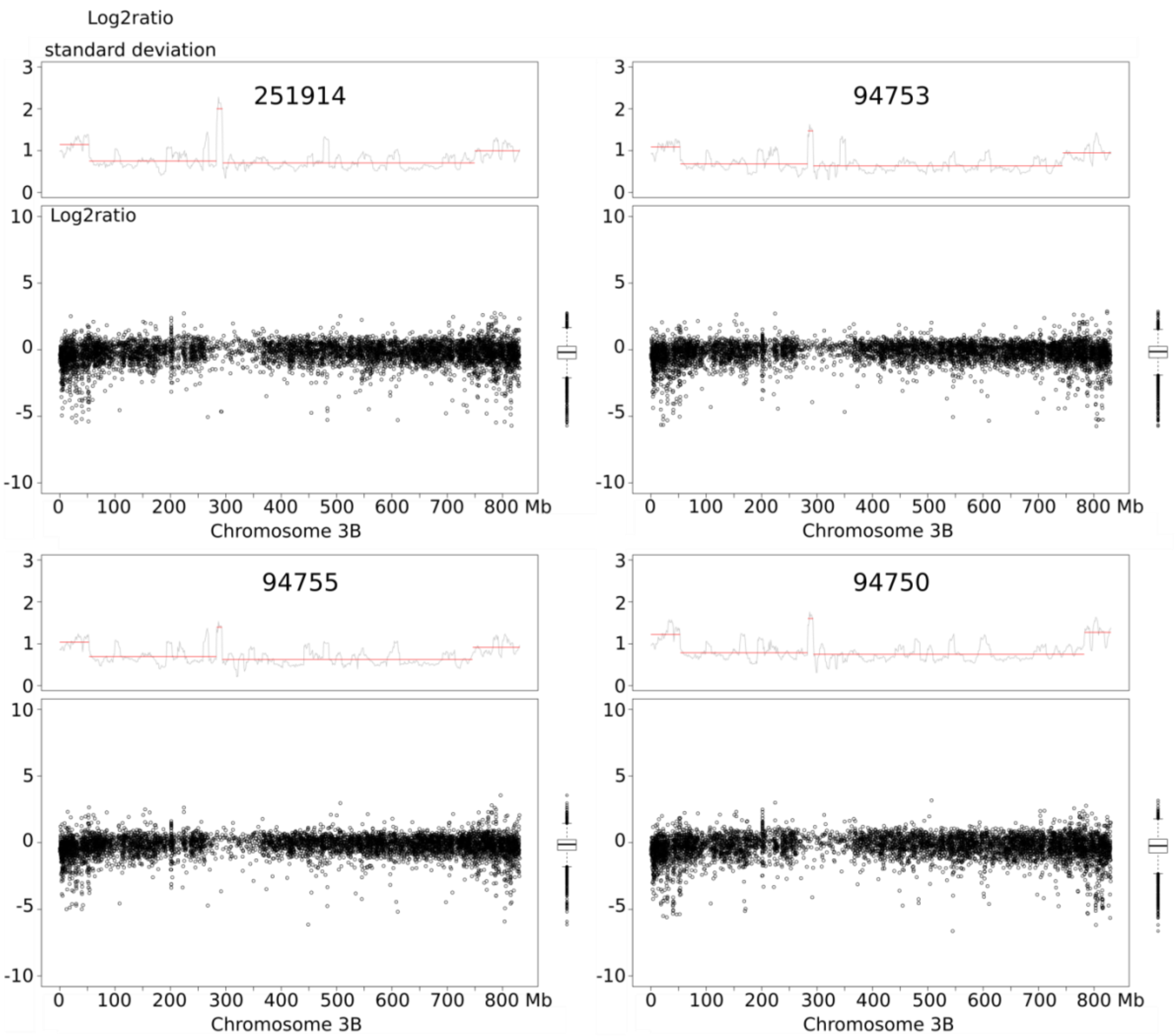

Figure S14: DoC log2ratios for *T. carthlicum* accessions.
DoC log2ratios of all genes across chromosome 3B and standard deviation calculated in a 10 Mb sliding window (step=1 Mb, R package changepoint, penalty: "BIC", penalty value: 0.05, method: “SegNeigh"). A boxplot of the DoC log2ratios with upper and lower whiskers considered as thresholds to define up and downCNVs is represented on the right.

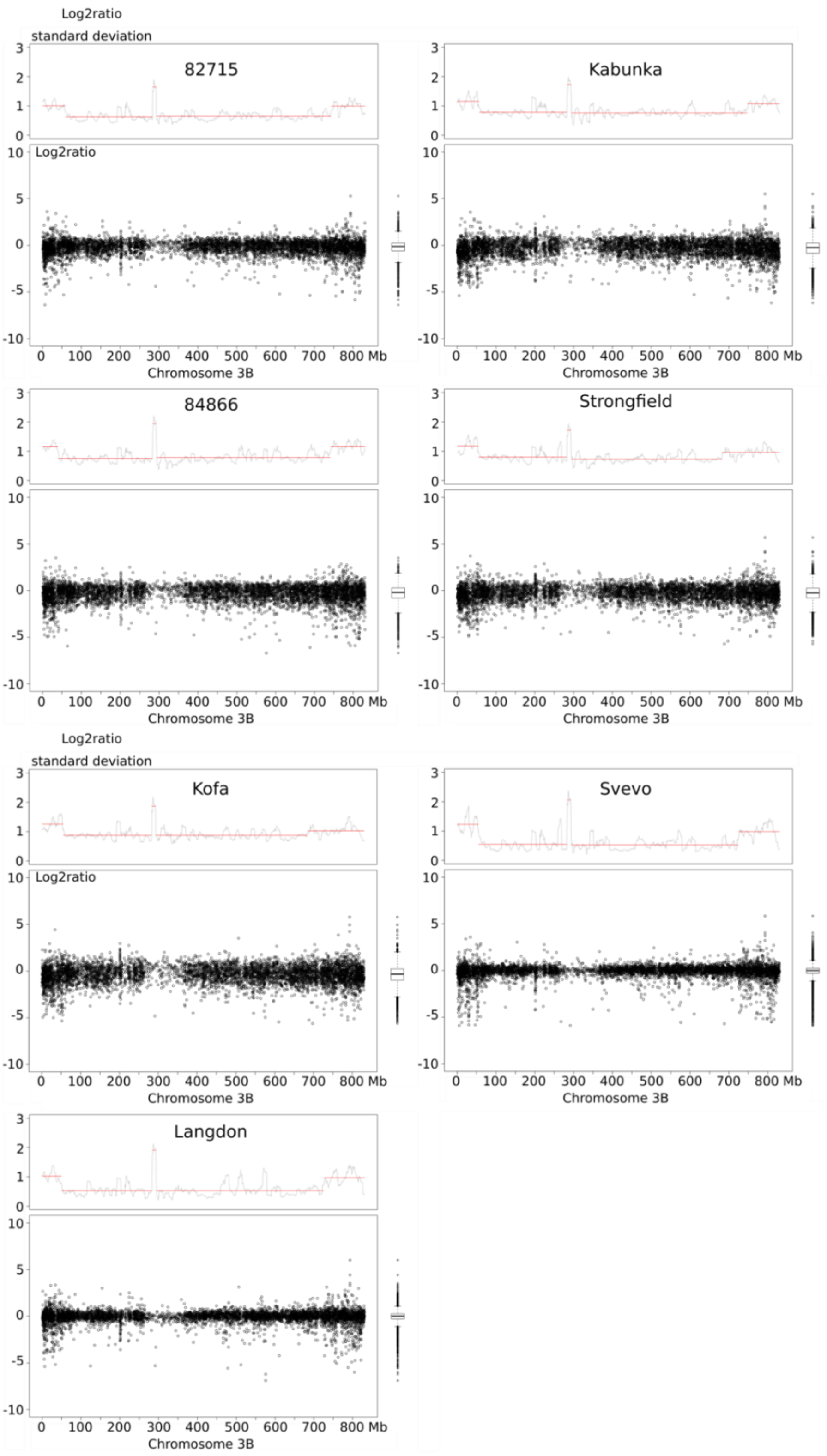

Figure S15: DoC log2ratios for *T. durum* accessions.

DoC log2ratios of all genes across chromosome 3B and standard deviation calculated in a 10 Mb sliding window (step=1 Mb, R package changepoint, penalty: "BIC", penalty value: 0.05, method: “SegNeigh"). A boxplot of the DoC log2ratios with upper and lower whiskers considered as thresholds to define up and downCNVs is represented on the right.

Table S1: Proportions (in %) of reads mapped on chr3B, chr2B, ChrUn, and all other chromosomes (Mapq>11) for the 45 resequencing datasets

Species (ploidy)	Accession name	Chr3B	Chr2B	ChrUn	Others
Tae (6x)	Chinese Spring	88	3	4	5
.	Sirazaiabarigi 2	88	2	2	7
.	Nanking_NO25	87	4	2	7
.	Fukuhokomugi	82	2	2	14
.	NZ(81)P43	86	2	1	10
.	M45/66	85	3	1	11
.	N67M2	85	4	1	10
.	Nachipundo	84	3	1	12
.	Oulianowska	88	2	2	9
.	Arche	87	2	2	9
.	Volt	86	2	2	11
.	Detenicka Cervena	87	2	1	9
.	Cerealor	88	2	1	9
.	Recital	87	2	2	9
.	Odessa Expsta21821	87	3	2	9
.	Redman	85	3	2	10
.	Marquis	86	2	1	11
.	Opata 85	83	3	1	13
.	Hope	75	10	1	14
.	Tom Thumb	83	3	1	12
Tma (6x)	24175	79	4	2	15
.	24174	83	4	2	11
.	24176	77	2	2	19
Tsp (6x)	EPEAUTRE 3 ROUX	81	2	1	16
.	EPEAUTRE DE L'AVEYRON	78	3	1	18
.	EPEAUTRE NOIR BARBU VELU	70	15	1	15
Tdi (4x)	45383	55	30	1	13
.	CWI17084	81	4	1	14
.	45309	78	6	1	15
.	415152	73	14	1	11
Tde (4x)	DD.100V	79	4	1	16
.	DD.PSEUDO-JORDANIC.61V	75	5	1	19
.	DD.SPONTANEO VILLOSUM 117V	75	4	1	20
Tca (4x)	251914	80	4	1	14
.	94753	82	3	2	13
.	94750	80	6	1	12
.	94755	64	11	1	24
Tdu (4x)	82715	88	2	1	9
.	KUBANKA	81	5	1	13
.	84866	85	3	1	11
.	Strongfield	85	4	1	11
.	Kofa	81	4	1	13
.	Langdon	87	4	1	8
.	Svevo	86	3	1	10
Synthetic (6x)	W7984 (Synthetic)	78	8	1	13

Table S2 :Number of ISBP inDels for each species

"Unique" indicates the number of inDels that are present in only one accession of the species. "Shared" and "All" indicate the number of inDels that are shared by at least 2 accessions and all accessions, respectively.

**Species**	**Unique**	**Shared**	**All**	**Total**
T. macha	16515	9517	33318	59350
T. aestivum	33251	109708	1129	144088
(East Asian)	*27098*	*12092*	*7327*	*46517*
(others)	*33995*	*96679*	*8920*	*139594*
T. carthlicum	13482	12771	38048	64301
T. dicoccum	40098	45335	22010	107443
T. dicoccoides	18375	16847	32928	68150
T. spelta	20357	17459	31539	69355
T. durum	32395	64238	19229	115862
**All accessions**	30781	155830	416	186611

Table S3: Number of SVs and newly discovered genes for each accession

Species (ploidy)	Accession name	No. PAVs	No. downCNVs	No. upCNVs	Total no. SVs	No. new genes
Tae (6x)	Sirazaiabarigi 2	86	58	22	166	26
.	Nanking_NO25	55	61	11	127	30
.	Fukuhokomugi	85	66	19	170	28
.	NZ(81)P43	151	92	19	262	40
.	M45/66	134	99	33	266	71
.	N67M2	52	68	21	141	68
.	Nachipundo	73	66	13	152	50
.	Oulianowska	105	67	16	188	50
.	Arche	155	82	19	256	40
.	Volt	120	70	19	209	74
.	Detenicka Cervena	42	73	19	134	52
.	Cerealor	147	92	26	265	57
.	Recital	121	84	29	234	46
.	Odessa Expsta21821	118	78	17	213	50
.	Redman	127	93	22	242	54
.	Marquis	150	66	8	224	43
.	Opata 85	134	74	22	230	55
.	Hope	158	63	7	228	34
.	Tom Thumb	138	80	15	233	55
Tma (6x)	24175	144	67	24	235	43
.	24174	149	62	24	235	46
.	24176	140	81	23	244	52
Tsp (6x)	EPEAUTRE 3 ROUX	169	87	23	279	49
.	EPEAUTRE DE L'AVEYRON	166	101	34	301	57
.	EPEAUTRE NOIR BARBU VELU	187	71	16	274	45
Tdi (4x)	45383	184	80	14	278	55
.	CWI17084	211	69	9	289	54
.	45309	208	75	11	294	26
.	415152	153	68	8	229	59
Tde (4x)	DD.100V	114	105	49	268	78
.	DD.PSEUDO-JORDANIC.61V	131	79	14	224	48
.	DD.SPONTANEO VILLOSUM 117V	124	91	30	245	53
Tca (4x)	251914	181	71	15	267	50
.	94753	187	76	19	282	43
.	94750	168	72	11	251	39
.	94755	184	79	20	283	41
Tdu (4x)	82715	172	78	29	279	56
.	KUBANKA	179	89	17	285	44
.	84866	217	75	16	308	49
.	Strongfield	189	85	24	298	50
.	Kofa	196	84	11	291	41
.	Langdon	152	109	45	306	48
.	Svevo	148	116	40	304	47
.	W7984 (Synthetic)	167	87	29	283	45
